# *RNU12* inhibits gastric cancer progression via sponging miR-575 and targeting BLID

**DOI:** 10.1038/s41598-023-34539-4

**Published:** 2023-05-09

**Authors:** Shaoli Wang, Changyan Zou, Xinyi Lin, Dan Hu, Ying Su, Huocong He, Xiongwei Zheng, Lurong Zhang, Tao Huang, Jin-rong Liao, Xiandong Lin

**Affiliations:** 1 Laboratory of Radiation Oncology and Radiobiology, Clinical Oncology School of Fujian Medical University and Fujian Cancer Hospital, Fuzhou, 350014 China; 2 Fujian Medical University, Fuzhou, 350122 China; 3 Department of Pathology, Clinical Oncology School of Fujian Medical University and Fujian Cancer Hospital, Fuzhou, 350014 China; 4 Shanghai Institute of Nutrition and Health, Chinese Academy of Sciences, Shanghai, 200031 China; 5 Fujian Provincial Key Laboratory of Translational Cancer Medicine, Fuzhou, 350122 China

**Keywords:** Cell biology, Biomarkers, Medical research, Cancer, Gastrointestinal cancer, Metastasis, Oncogenes, Tumour biomarkers, Tumour-suppressor proteins

## Abstract

Gastric cancer (GC) is one of the major causes of cancer deaths with 5-year survival ratio of 20%. *RNU12* is one of long noncoding RNAs (lncRNAs) regulating the tumor progression. However, how RNU12 affecting GC is not clear. qRT-PCR was utilized for determining the *RNU12* expression in cell lines, 113 cases of paired gastric cancer (GC) and their adjacent normal gastric tissues. The biofunction alterations of *RNU12* were assessed by its overexpression or knockdown in GC cells. MTT and cloning assay were assayed for the cell proliferation, the flow cytometry for the detection of cell cycle and the wound healing assay (WHA) and transwell invasion assay (TIA) for examining the migration and invasion of cells. The expressions of a set of genes related proliferation and migration were investigated with the Western Blotting (WB). RNA immunoprecipitation (RIP), biotinylated RNA pull-down and dual luciferase reporter tests were used to detect the interactions of *RNU12* with miR-575/BLID. The in vivo proliferation and migration ability of *RNU12* infected cells were determined in zebrafish system. This study revealed that *RNU12* inhibited proliferation, invasion and metastasis by sponging of miR-575 and regulating the downstream BLID and modulated EMT of GC cells. The *RNU12*/miR-575/BLID axis is likely to be the prognosis biomarkers and drug targets of GC.

## Introduction

Gastric cancer (GC) is a common type of cancer worldwide, ranking the third of cancer death following lung cancer and liver cancer^1^. Due to the early stages of GC are clinically silent, the most patients were clinically diagnosed at the advanced stage. For this reason, its 5-year survival rate (SR) < 20%^2^. Although statistics show a gradual decrease of GC incidence rates, an unexplained increase in GC incidence of younger individuals from developed regions is emerging^3^. In addition, risk factors for gastric cancer are diverse^4^; Although the primary cause is considered to directly related to *Helicobacter pylori*^4,5^. Previous findings showed that tobacco use, familial predisposition, alcohol consumption, pernicious anemia, high dietary salt consumption, previous gastric surgery, processed meat consumption, age, and low socio-economic status are all associated with the occurrence of GC^6–10^. The complexity of the GC causes promotes us to understand the molecular mechanisms behind the GC, which could lead to advancements in diagnosis and treatment.

Long noncoding RNAs (lncRNAs) are usually considered to be a length over 200 nt RNA and can not code protein^11^. LncRNAs feature abundant quantity, diversity, and action mechanisms related with various biological cancer processes, such as carcinogenesis, apoptosis, differentiation, proliferation, invasion, metastasis, etc.^12,13^. The lncRNAs exert the function of “sponges” chelating miRNAs from target mRNAs to antagonize the biological function possessed by miRNAs as competitive endogenous RNAs (ceRNAs)^14^. For example, SNHG8 as a lncRNA presents an abnormal upregulation in GC and sponges to the miR-5125-p for promoting GC cell invasion^15^. LncRNA ZNF667-AS1 sponges miR-1290 and promotes ABLIM1 expression to suppress nasopharyngeal cancer (NPC)^16^. Some evidence has indicated that closely associations between expression levels of lncRNAs in GC tissues^17^. Accumulating evidence has shown that lncRNAs play a critical role in the progress of GC.

*RNU12* is in the 22q12-q13 chromosomal region, and associated with many diseases, such as psoriatic arthritis (PSA)^18^, the MOPD I phenotype^19,20^, CDAGS Syndrome^21^ and congenital cerebellar ataxia^22^. Recent study indicates knockdown of lnc-*RNU12* can influence cell cycle by altering the expression of protein-coding genes related to the cell cycle and apoptosis in immune T cells^23^. However, the relationship between *RNU12* and GC progression has been poorly studied. Our previous results have discovered that *RNU12* was a decreased expression level in GC, suggesting that *RNU12* is an essential regulator in suppressing tumor genesis. Here, we explored the function of *RNU12* in controlling the tumor growth and progression and found that *RNU12*, as a sponge of miR-575, reduced miR-575 expression, regulated *BLID* expression and reduced GC cell proliferation and metastasis.

## Results

### Characterization of *RNU12* in GC and its association with clinicopathological features in GC patients

**Figure 1:**
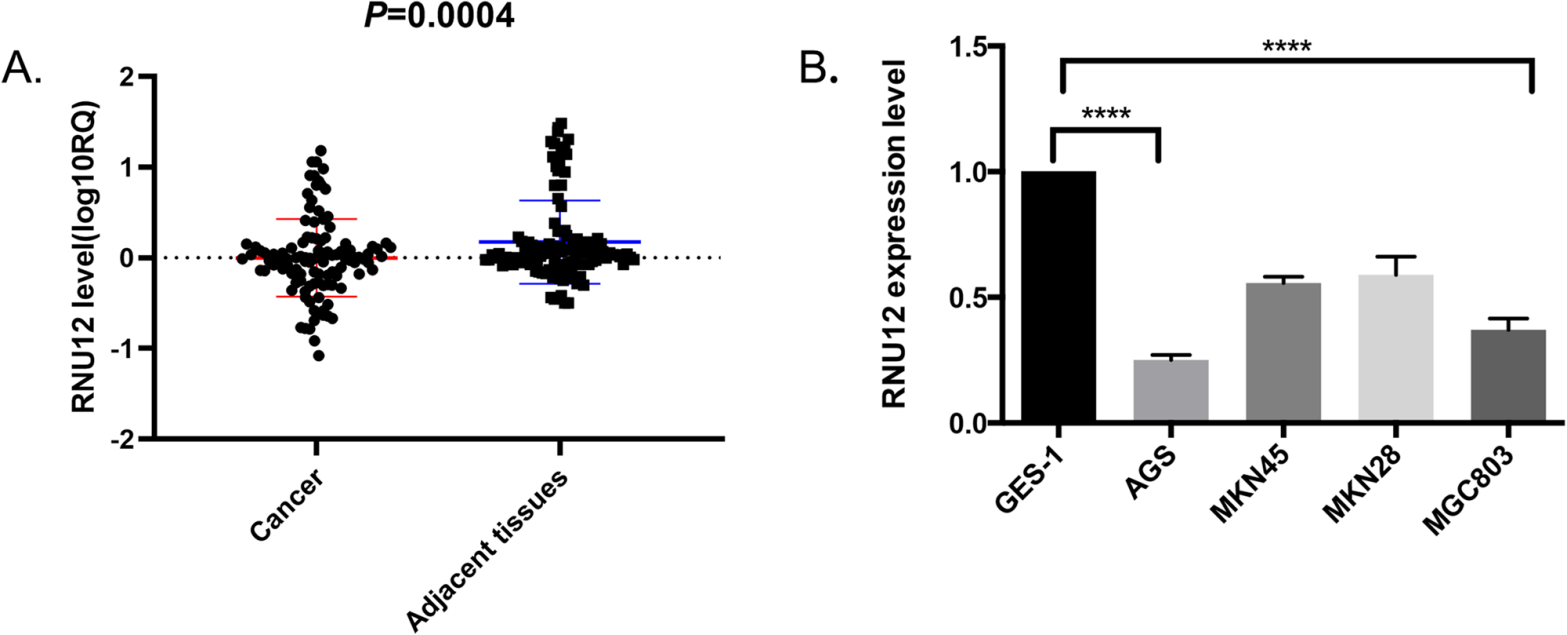
*RNU12* was down-regulated in GC. (**A**) Expression of *RNU12* in 113 paired GC and adjacent tissues; The data is analyzed to conform to a normal distribution and presented as the mean ± SD value (N = 113), y-axis units (log10RQ, RQ = 2^−ΔΔCt^). (**B**) Expression of *RNU12* in GES-1 and 4 human GC cell lines (AGS, MKN45, MKN28 and MGC803). *P < 0.05; **P < 0.01; ***P < 0.001; ****P < 0.0001.

The real-time PCR detected the expression level of *RNU12* in 113 cases of GC and their adjacent tissues. The *RNU12* expression in GC tissues was 0.0008 ± 0.428 (log10RQ, RQ = 2^−ΔΔCT^) while that of the corresponding adjacent tissue was 1.744 ± 0.458 (P = 0.001, Fig. 1A). Similarly, a real-time PCR assay was performed in 1 normal human gastric mucosa cell line (Ges-1) and 4 GC tumor lineage cells (AGS, MKN45, MKN28 and MGC803). The result (Fig. 1B) showed that the much lower RNU 12 expression in the 4 GC tumor cell lines than that in normal gastric cell line, Ges-1.

The clinical data demonstrated a negative relationship between *RNU12* expression and clinic pathologic feature-lymph node metastasis in GC patients (Table 1, P < 0.05).

**Table 1:** Relationship of *RNU12* expression with clinicopathologic features.

Clinicopathologic feature	Case	Expression levels(lgRQ)	*P*-value
Tumor size (cm)			0.646
< 5 cm	56	0.018 ± 0.408	
≥ 5 cm	57	− 0.019 ± 0.449	
Invasion depth			0.193
< T2	10	0.168 ± 0.334	
≥ T2	103	− 0.017 ± 0.434	
Lauren typing			0.544
Intestinal type	77	− 0.176 ± 0.445	
Diffuse type	36	0.035 ± 0.391	
TNM stage			0.070
I + II	39	0.099 ± 0.435	
III + IV	74	− 0.053 ± 0.417	
Lymph node metastasis			0.011*
Non-metastasis	30	0.167 ± 0.439	
Metastasis	83	− 0.061 ± 0.409	

*P < 0.05.

### Overexpression of *RNU12* inhibits GC proliferation, migration, and invasiveness

**Figure 2:**
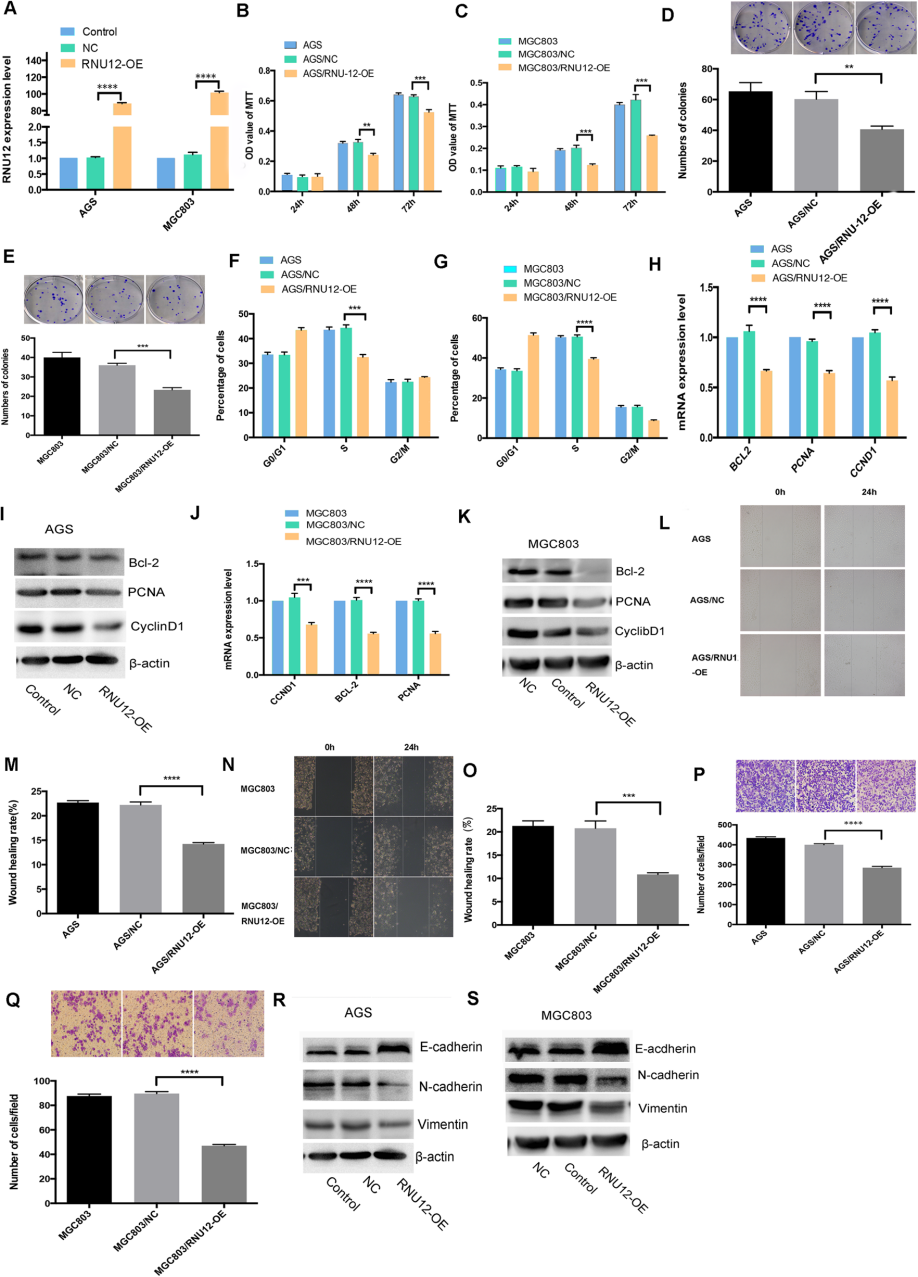
*RNU12* overexpression inhibited GC cells in terms of the proliferation, migration, and invasion. (**A**) Real-time PCR on RNU 12 expression in AGS and MGC 803 cells; (**B**,**C**) The impact of RNU 12 overexpression on the cell vitality under the measurement of MTT assay; (**D**,**E**)The impact of RNU 12 overexpression on cell colony formation; (**F**,**G**) The impact of RNU 12 overexpression on cell cycle distribution under FACS; (**L**–**O**) The impact of RNU 12 overexpression on cell migration under WHA; (**P**,**Q**) The impact of RNU 12 overexpression on cell invasiveness under transwell invasive assay (TIA); (**H**–**K**) qRT-PCR and WB detected the expressions regarding proliferation-related-genes of *Bcl-2*, *CCND1*, and *PCNA*; (**R**,**S**) The expression exhibited by invasion/metastasis-related-genes, *E-cadherin*, *VIMENTIN* and *N-cadherin* according to WB. *P < 0.05; **P < 0.01; ***P < 0.001; ****P < 0.0001.

To determine the way if *RNU12* overexpression affected GC behaviors, AGS and MGC803 cell lines were infected with HBLV-NC or HBLV- RNU12-OE lentivirus, respectively. RT-qPCR analysis illustrated the obvious increase in *RNU12* expression in RNU12-OE relative to NC in AGS or MGC803 cell lines (Fig. 2A). MTT assay (Fig. 2B,C) and colony assay (Fig. 2D,E) demonstrated the overexpression of *RNU12* reduced the cell proliferation and colony formation. In addition, S phase of cell cycle was remarkably decreased in the *RNU12* overexpressed AGS or MGC803 cells as compared to control AGS/NC or MGC803/NC cells (Fig. 2F,G). The results of WHA and TIA showed that upregulation of *RNU12* weakened the capacities of wound healing (Fig. 2L–O) and the transwell invasiveness (Fig. 2P,Q). Furthermore, the results of RT-qPCR and WB indicated that there were downregulations of development-related genes (*CCND1*, *PCNA*), EMT-associated genes (*N-cadherin*, *Vimentin*) and anti-apoptotic *BCL-2*, while *E-cadherin* was upregulated in AGS/RNU12-OE or MGC803/RNU12-OE cells (Fig. 2H–K,R,S); These findings showed that *RNU12* might inhibit the GC cell aggressiveness in vitro (the original blots in Fig. 2I,K,R,S are shown in Supplementary file 1).

### *RNU12* silencing facilitates GC proliferation, migration, and invasiveness

**Figure 3:**
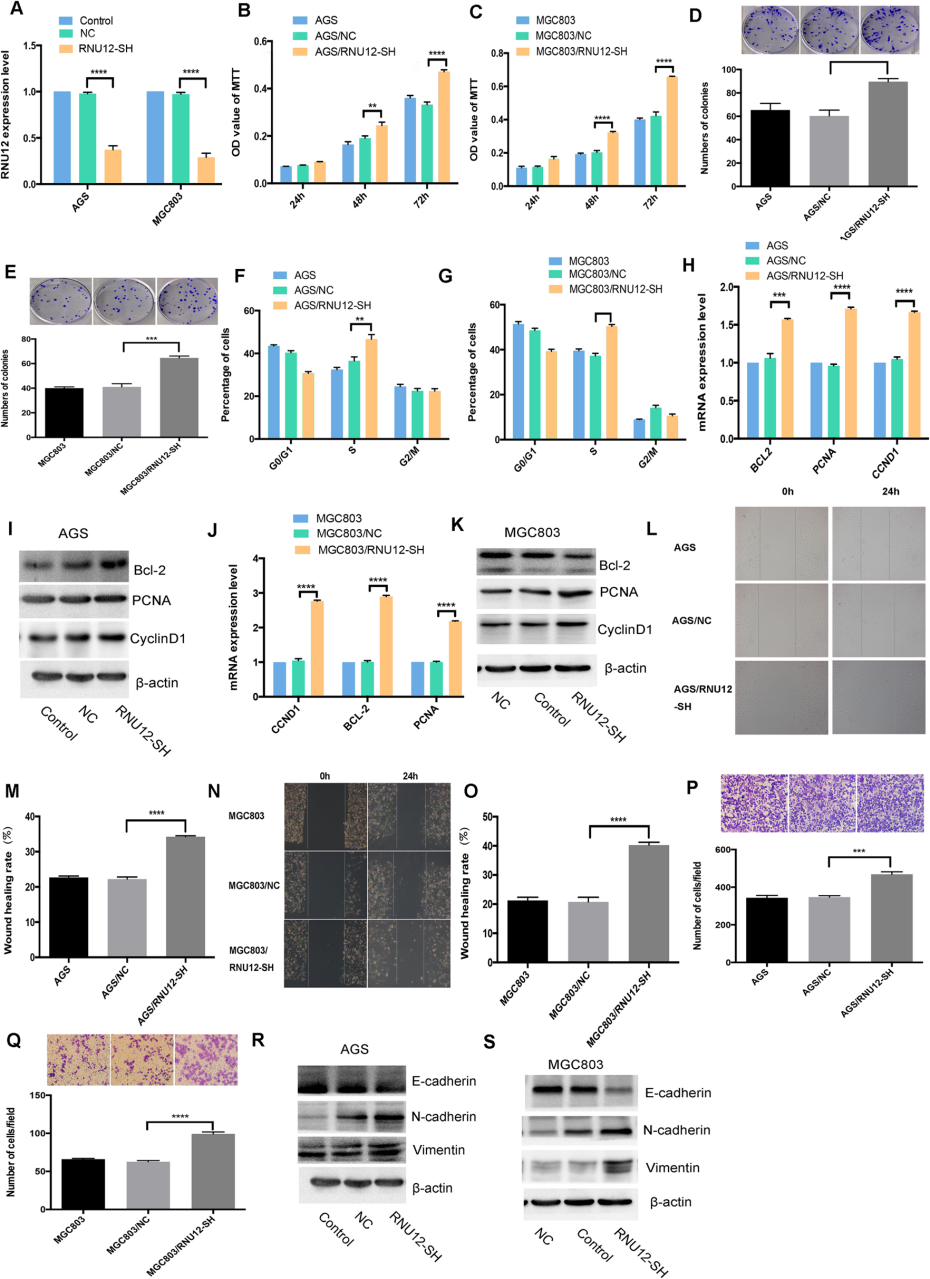
*RNU12* silencing enhanced the proliferation, migration, and invasiveness of AGS and MGC 803 cells. (**A**) Real-time PCR on *RNU12* expression in the two types of cells; (**B**,**C**) The impact of *RNU12* silencing on various AGS and MGC 803 cells under the measurement of MTT assay; (**D**,**E**) The impact of *RNU12* silencing on cell colony formation; (**F**,**G**) The impact of *RNU12* knockdown on the cell cycle distribution under FACS; (**L**–**O**) The impact of *RNU12* silencing on cell migration under WHA; (**P**,**Q**) The impact of *RNU12* silencing on cell invasiveness under TIA; (**H**–**K**) qRT-PCR and WB detected the expression exhibited by *BCL-2*, *CCND1*, and *PCNA*; (**R**,**S**) WB confirmed the expression regarding EMT-associated genes, *E-cadherin*, *VIMENTIN* and *N-cadherin*. *P < 0.05; **P < 0.01; ***P < 0.001; ****P < 0.0001.

For evaluating whether silencing of *RNU12* could enhance GC malignant behaviors, AGS and MGC803 were transfected with HBLV-NC or HBLV-RNU12-SH lentivirus, respectively. Based on RT-qPCR analysis, the above treatments could remarkably decrease the *RNU12* expression in RNU12-SH compared with NC in AGS or MGC803 cell lines (Fig. 3A). *RNU12* silencing significantly promotes AGS or MGC803 in terms of the proliferation and the colony formation (Fig. 3B–E). In addition, relative to AGS/NC or MGC803/NC, an obvious increase was observed in the number of cells arrested at the S phase of AGS/RNU12-SH or MGC803/RNU12-SH as measured by a Flow Cytometer (Thermo Fisher Scientific, USA), (Fig. 3F,G). Furthermore, downregulation of *RNU12* enhanced the wound healing migratory capacity and the transwell invasiveness (Fig. 3L–Q). The results of RT-qPCR and WB showed the upregulation of proliferation-related genes (*CCND1*, *PCNA*), EMT-associated genes (*N-cadherin*,*Vimentin*) and anti-apoptotic *BCL-2*, while downregulation of E-cadherin in AGS/RNU12-SH cells (Fig. 3H–K,R,S). The above findings indicated that the ability of *RNU12* silencing could enhance the proliferation, migration, and invasiveness of GC cells in vitro (the original blots in Fig. 3I,K,R,S are shown in Supplementary file 2).

### *RNU12* sponges hsa-miR-575 and regulates BLID

**Figure 4:**
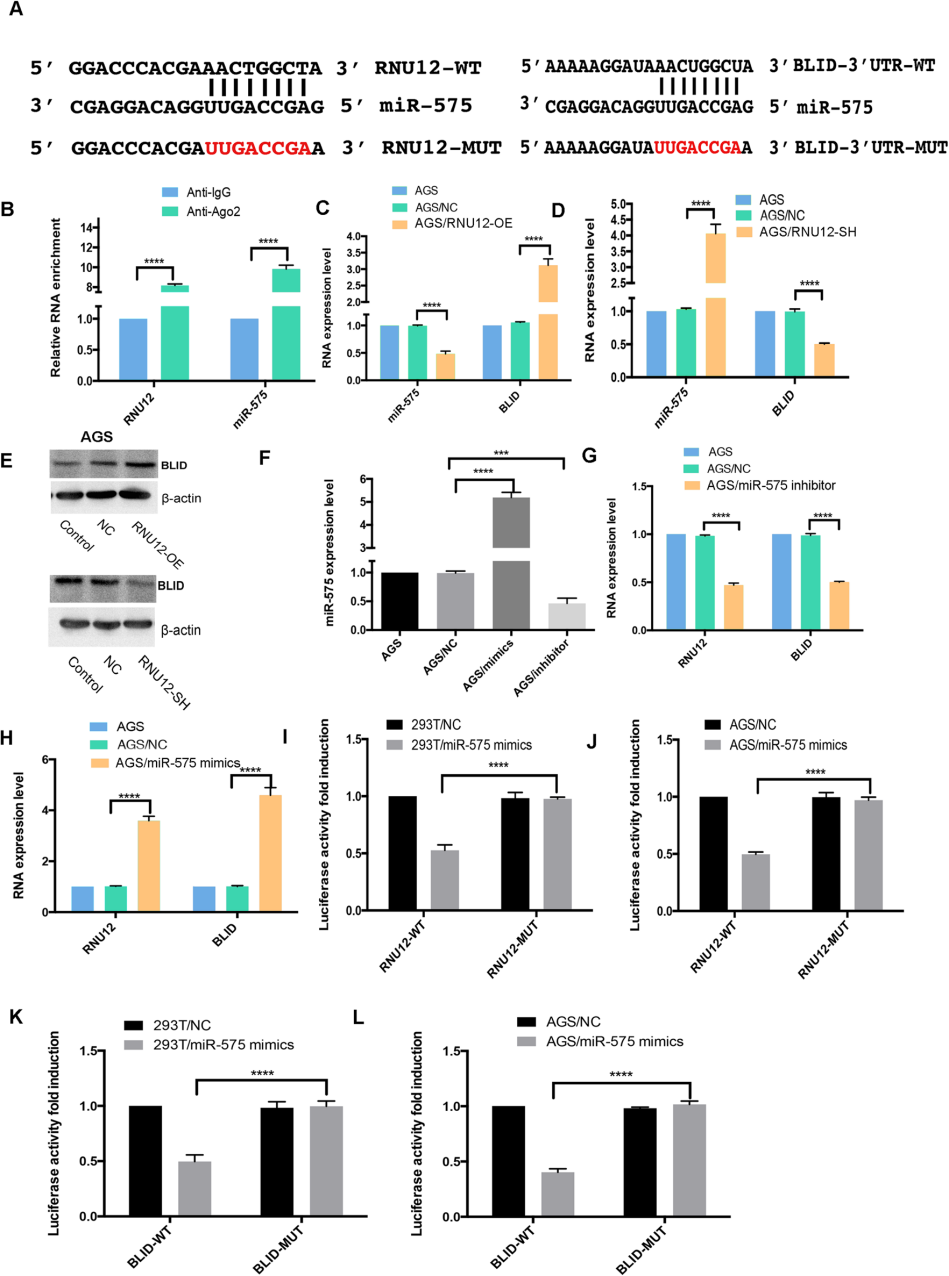
*RNU12* sponged hsa-miR-575 and regulated *BLID*. (**A**) Sequence alignment of hsa-miR-575 which had putative binding sites in *RNU12* and *BLID* wt and mt regions; (**B**) After detecting ago2 or IgG RIP assay, qRT-PCR assisted in determining the *RNU12* and miR-575 levels; (**C**,**D**) MiR-575 expression presented a decrease and increase in AGS /RNU 12-OE cells and AGS /RNU 12-SH cells under qRT-PCR, respectively; (**C**-**E**) AGS /RNU 12-OE and AGS /RNU12-SH cells presented increased and decreased *BLID* expression under qRT-PCR and WB, respectively; (**F**) qRT-PCR analyzed the miR-575 expression in AGS cells receiving the transfection of miR-575 mimics or inhibitors; (**G**,**H**) qRT-PCR analyzed *RNU12* and *BLID* expression in AGS cells receiving the transfection of miR-575 mimics or inhibitors. (**I**–**L**) Based on Double Luciferase Report analysis, hsa-miR-575 mimics weakened the fluorescence intensity exhibited by 293 T or AGS cells under the transfection of RNU12-Wt or BLID-Wt rather than RNU12-Mut or BLID-Mut vector. *P < 0.05; **P < 0.01; ***P < 0.001; ****P < 0.0001.

The crosstalk between Marianas and *RNU12* in GC cells was detected, as lncRNAs mainly played a role as miRNA spongers^24,25^. We used the lncRNASNP2 database to conclude that hsa-miR-575 is a potent target of *RNU12*. Using the TargetScan and miRDB databases, we further found that *BLID* was also a potent target specific to hsa-miR-575. Predicted binding sites regarding hsa-miR-575 and wt regions of *RNU12* and *BLID* were presented in DNA sequences (Fig. 4A). RIP assay revealed the co-enrichment of *RNU12* and hsa-miR-575 in a manner depending on Ago2 (Fig. 4B). RT-qPCR analysis revealed the ability of *RNU12* overexpression or silencing to remarkably lower or improve hsa-miR-575 expression in AGS (Fig. 4C,D), respectively. In addition, according to results of RT-qPCR and WB, the expressions of *BLID* compared to hsa-miR-575 were positively correlated with that of *RNU12* (Fig. 4C–E). Parallelly, hsa-miR-575 overexpression lowered the *RNU12* and *BLID* expressions. Consistently, hsa-miR-575 knockdown contributed to an increase in *RNU12* and *BLID* expressions (Fig. 4F–H). The dual-luciferase reporter assay was used to analyze whether *RNU12* and *BLID* were functional targets specific to hsa-miR-575. The results showed that the luciferase activity presented an obvious decline in that hsa-miR-575 mimics were transfected into cultured RNU12-Wt or BLID-Wt-co-transfected HEK293T and AGS cells. However, the same cell receiving the transfection of RNU12-Mut or BLID-Mut vector had no effect (Fig. 4I–L). The integral results suggested that *RNU12* and *BLID*’s 3′ UTR possessed hsa-miR-575-binding sites. Hsa-miR-575 acted as the *RNU12* inhibitor and the *BLID* blocker in the GC progression. (The original blots in Fig. 4E are shown in Supplementary file 3).

### BLID enhancement critically affects malignant behaviors mediated by *RNU12*

**Figure 5:**
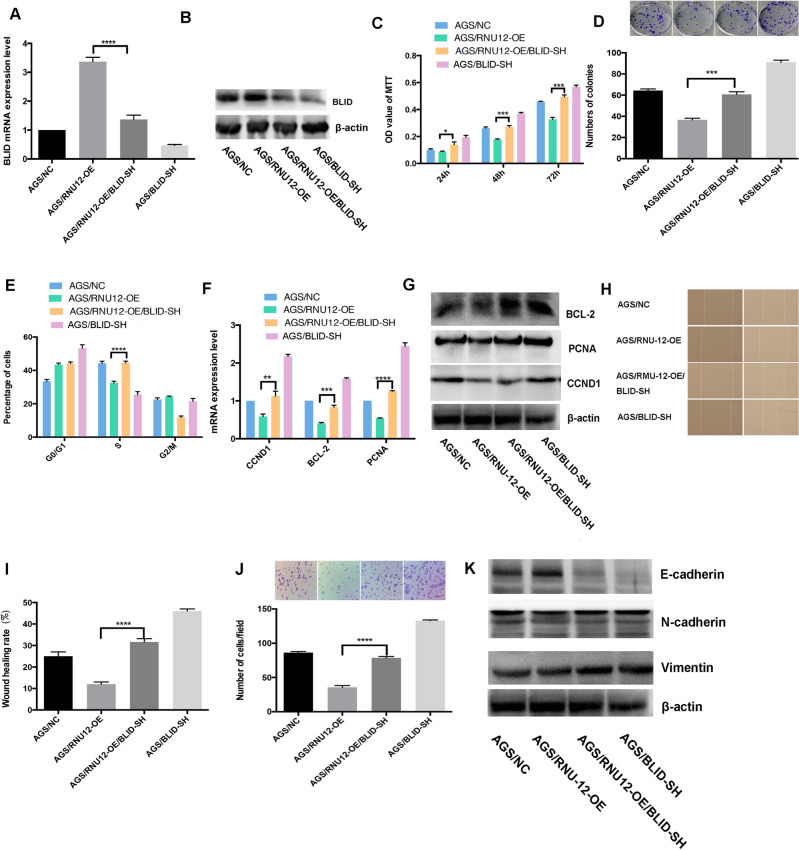
*BLID* enhancement critically inhibited malignant behaviors mediated by *RNU12*. (**A**,**B**) *BLID* expression in AGS cells in qRT-PCR and WB rescue assay. (**C**,**D**) MTT assay and colony formation regarding AGS cells. (**E**) AGS cell cycle distribution by FACS; (**H**,**I**) WHA of AGS cells; (**J**) TIA of AGS cells; (**F**,**G**) The expression of genes related to proliferation: *BCL-2*, *CCND1*, and *PCNA* under the measurement of qRT-PCR and WB; (**K**) The expressions of genes related to EMT: *E-cadherin*, *N-cadherin*, *VIMENTIN* based on WB. *P < 0.05; **P < 0.01; ***P < 0.001; ****P < 0.0001.

For investigating whether *BLID* attenuated malignant behaviors mediated by *RNU12*, *BLID* was silenced in AGS /RNU12-OE in a rescue assay. The qRT-PCR and WB showed that AGS/RNU12-OE/BLID-SH presented obviously lower *BLID* compared to AGS/RNU12-OE (Fig. 5A,B). The silencing of *BLID* could enhance the proliferation and the colony formation in AGS/RNU12-OE cells determined by MTT assay (Fig. 5C) and colony assay (Fig. 5D). By silencing *BLID*, the cell cycle arrest was remarkably enhanced at the S phase of AGS compared to AGS/RNU12-OE (Fig. 5E). In addition, as revealed by qRT-PCR and WB assay, silence of *BLID* in AGS/RNU12-OE cells elevated the expression of genes associated with proliferation or apoptosis, (*BCL-2*, *CCND1*, and *PCNA*) (Fig. 5F,G). The silence of *BLID* also assisted in remarkably increasing the migratory and invasive abilities exhibited by AGS/RNU12-OE cell lines, based on the WHT (Fig. 5H,I) and TIA (Fig. 5J). The results of qRT-PCR and WB showed an upregulation of downstream molecules (*N-cadherin*, and *Vimentin*) related to metastasis, and the downregulation of *E-cadherin* (Fig. 5K). Taken together, silencing *BLID* critically affects the malignant behaviors mediated by RNU12 in GC cells in vitro (The original blots in Fig. 5B,K are shown in Supplementary file 4).

### *RNU12* promotes growth of GC tumor in zebrafish

**Figure 6:**
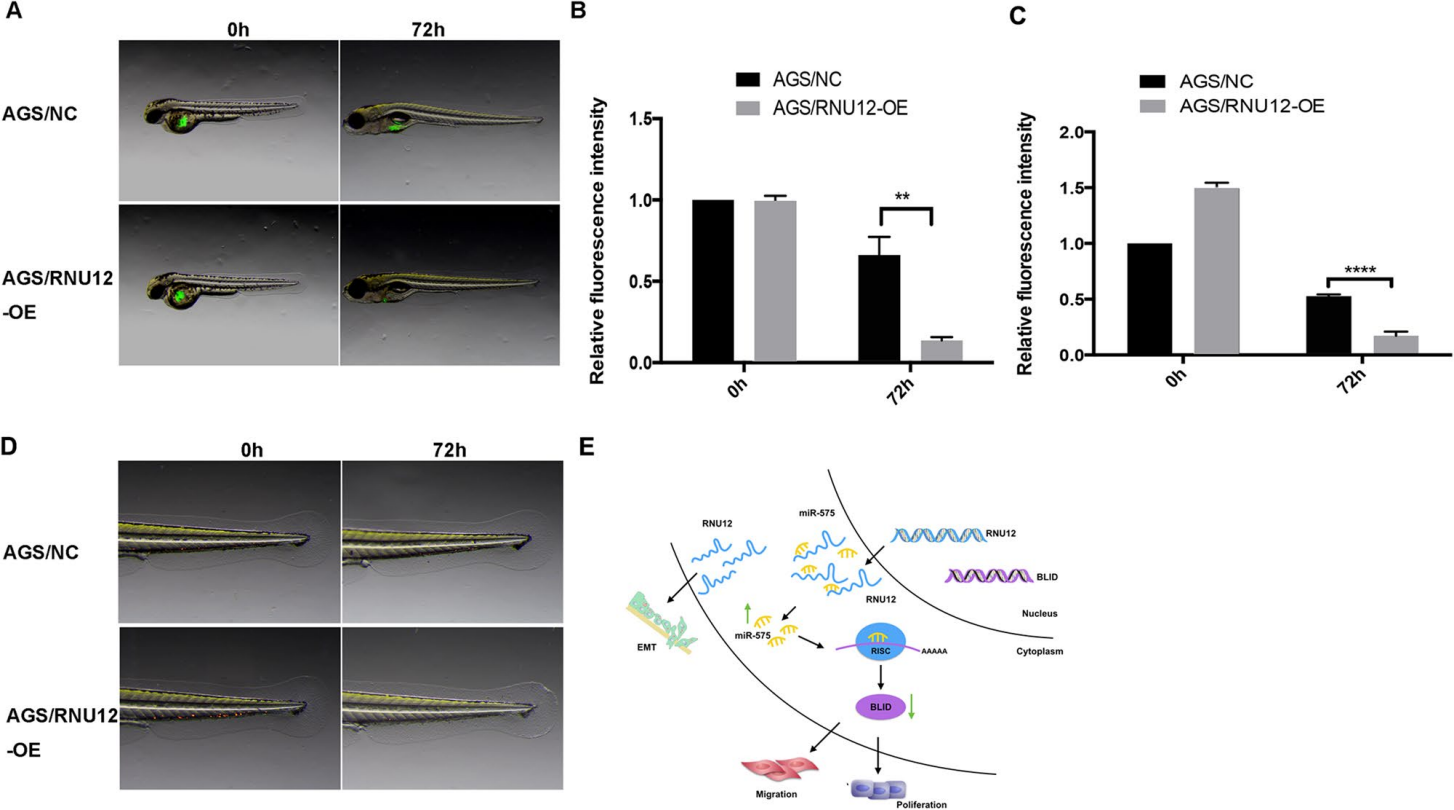
*RNU12* inhibited GC tumor growth in zebrafish. (**A**) Observation of fluorescence distribution exhibited by GC cells with GFP in whole body by stereomicroscope and confocal microscope at various time points; (**B**) The fluorescence area of whole body of GC cells at various time points; (**C**) The GC cell tail fluorescence area at various time points; (**D**) Observation of the fluorescence distribution exhibited by GC with GFP in tail by stereomicroscope together with confocal microscope at various time points; (**E**) Schematic diagram of the mechanism: *RNU12* down-regulation in gastric cancer cell modulates EMT and acts as a miR-575 sponge to promote its function and down-regulates *BLID*, forming RNU12/miR-575/BLID axis to promote progression and migration of gastric cancer. *P < 0.05; **P < 0.01; ***P < 0.001; ****P < 0.0001.

The GC tumor xenograft in zebrafish model was used for exploring the association of the *RNU12* biological function in vivo. The experiment (Fig. 6A,B) on the zebrafish that received AGS cell injection at various time points traced the fluorescence distribution exhibited by GC cells in vivo with GFP (Green fluorescent protein). The results revealed the obviously decreased GFP-AGS/RNU12-OE cells (*P* < 0.001) relative to the AGS/NC in zebrafish at 72 h, indicating that the overexpression of *RNU12* attenuated GC cells’ proliferation in vivo as that in vitro. In addition, AGS/RNU12-OE possessed lower tail GFP area relative to AGS/NC at 72 h, indicating that the overexpression of *RNU12* inhibited migration of GC cell (*P* < 0.001, Fig. 6C,D). All above results demonstrated the inhibitory effects of *RNU12* on GC proliferation, migration, and invasiveness in vivo.

This study revealed the gastric cancer-associated lncRNA *RNU12* is a tumor-suppressor lncRNA that inhibits cancer progression via miR-575/BLID axis, which plays crucial role in gastric cancer progression and suggest that *RNU12* is potential GC marker and target for GC therapy.

## Discussion

LncRNAs affect cancer progression^26–29^, However, the effects of *RNU12* on GC are unreadable^21,22^. In this study, we revealed for the first time the function of *RNU12* and its related molecules in GC: (1) *RNU12* was downregulated in GC with negative relation to lymph node metastasis; (2) *RNU12* could sponge miR-575 and upregulate *BLID*, then *BLID* functioned as a tumor suppressor; (3) *RNU12* could control proliferation, colony formation, migratory, invasion through RNU12/miR-575/BLID axis; (4) *RNU12* exerted the tumor-suppressing effect via regulating the expression regarding *CCND 1*, *PCNA*, *N-cadherin*, *E-cadherin*, *Vimentin* and anti-apoptosis *BCL-2*. Our results aid to the comprehensive understanding of the *RNU12* functionality (Fig. 6E).

LncRNAs are a species of ncRNA, with the length usually over 200 nt and having tissue-specific expression as exonic, intronic, overlapping, and intergenic^30,31^. Basic and clinical researchers have focused on comprehensively investigating the adoption of lncRNAs as new biomarkers and proper candidates for different cancer types^32^. Evidences indicated that lncRNAs could regulate the expression of gene at various genomic, transcriptomic and post transcriptomic levels, and could serve as the putative biomarkers and the therapeutic targets for GC^31,33^. LINC00589 promoted hnRNPA1 protein ubiquitination and proteasomal degradation for suppressing tumor progression, meanwhile restricting the peritoneal metastasis in GC, hence achieving the suppression of tumors^34^. LncRNA ZFAS1 modulated LIN28 and CAPRIN1 expression for enhancing the invasion and proliferation of GC cells, which suggested the possibility of ZFAS1 being a marker for GC diagnosis and prognosis^35^. Our present data supported the function of *RNU12* in inhabiting GC tumor progression through suppressing the cell growth, migration, invasion, as well as the proliferative ability expression with *CCND1*, *PCNA*, *N-cadherin*, *E-cadherin*, *Vimentin* and *BCL-2*.

Clearly, lncRNA competitively bound to proper miRNA to sponge miRNA, and prevented miRNA targeting mRNA for weakening the gene suppression mediated by miRNA^36–38^, regulating cellular proliferation, migration, and angiogenesis in cancer^38,39^. Our results confirmed that *RNU12* sponged miR-575p with evidences: (i) endogenous *RNU12* enriched in the AGO2-FLAG IP fraction and could possibly incorporate into RISC complexes (silencing complex induced by RNA); (ii) RNA pull-down assays revealed the enrichment of *RNU12* in the miR-575 biotin IP fraction; (iii) luciferase reporter assays demonstrated the binding of *RNU12* with miR-575; (iv) miR-575 knockdown elevated *BLID* expression; and (v) miR-575 over-expression inhibited *RNU12* and *BLID* expression. Our data demonstrated that *RNU12* could sponge miR-575 and weaken miRNA mediated gene *BLID* function.

MicroRNAs (miRNAs) refer to a series of non-coding RNAs and can remarkably help to regulate protein expressions^40^. The previous reports have confirmed the miRNA profiles can reflect differentiation state of the tumor and regulate mRNA interpretation^41^. MicroRNA-575 was first reported in 2009^42^. MiR-575 is oncogene in many tumors. And miR-575 targets PTEN for regulating the proliferation and apoptosis of cancer cells, thereby critically affecting GC^43^. Furthermore, the regulatory relationship between miR-575 and *BLID* has been reported in NSCLC^44^, which is consistent with our results defined in GC for first time.

BLID is a BH3-like motif that contains apoptotic member belonging to the Bcl-2 family. According to previous studies, *BLID* could suppress tumor and was associated with tumor prognosis^45,46^. *BLID* downregulated AKT pathway for inhibiting the cell growth and metastasis of breast cancer^47^. *BLID* could also assist in independently predicting the prognosis of breast cancer in terms of the overall survival and the distant metastasis-free survival^48,49^. In our study, we indicated *BLID* suppressed cell proliferation and invasion of GC. We first confirmed that miR-575 could regulate the expression of *BLID* in GC.

## Methods

### Tissue specimen collection

113 pairs of GC and their adjacent normal gastric tissues came from tumor biobank of Fujian Cancer Hospital. From June 2014 to June 2019. The patients undergone surgical treatment prior to any chemotherapy or radiotherapy in Fujian Cancer Hospital (Fuzhou, China). All samples were collected and analyzed after we obtained patients’ written informed consents. The Ethics Board of the Fujian Cancer Hospital (Fuzhou, China) approved all experiments. These fresh tissues were immediately submerged into liquid nitrogen and frozen at − 80 °C. The experiments were conducted following the Helsinki declaration.

### Cell lines and cell culture

Cell lines, including human GC cell lines: AGS, MGC803, MKN45, MKN28 and human normal gastric epithelial cell line GES-1, came from the Cell Bank of Type Culture Collection of the Chinese Academy of Sciences, confirmed by short tandem repeat DNA finger printing (STR) and free of mycoplasma contamination. The cell culture was performed in RPMI-1640 (GIBCO, Grand Island, NY, USA) with 100 IU/ml penicillin, 100 µg/ml streptomycin, 10% FBS (GIBCO) and with 5% CO_2_ at 37 °C.

### Construction of overexpression or knockdown *RNU12* cell lines

To perform experiments on *RNU12* overexpression or knockdown, the cells were infected with the virus vectors (Hanheng Biotechnology CoLtd). To establish the stable cell lines with overexpressed *RNU12*, HBLV-RNU12-OE (pHBLV-CMV-mcs-3flag-EF1-ZsGReen-T2A-PURO inserted with *RNU12* gene) was used and HBLV-NC as the vector alone control. Similarly, the virus vectors inserted with shRNA were used to establish stable *RNU12* knockdown cell lines, HBLV-RNU12-shrna1, HBLV-RNU12-shrna2, HBLV-RNU12-shrna3 and their parental vector pHBLV-U6-MCS-CMV-Zs/mcherry as the vector alone control. 10 MOI of viral vectors were used for infecting AGS or MGC 803 cells then seeded to six-well plates (5 × 10^5^/well) with 6 μg/ml of polyamine. 48 h later, the medium was added with puromycin (2 μg/ml). The cells were maintained in the above medium for 2 weeks for obtaining steady infected cells. The stable cell lines were named as AGS/NC, AGS/RNU12-OE, AGS/RNU12-SH, MGC803/NC, MGC803/RNU12-OE, MGC803/RNU12-SH. The symbol -OE represents overexpression and –SH represents knockdown. (The target-specific primer sets and primary antibodies are described in Supplementary Table S1).

### Overexpression BLID AGS cell construction

AGS cells in 5 × 10^5^/well plates underwent infection of 10 MOI of HBLV-BLID-Null-Zs Green-PURO virus vectors (Hanheng Biotechnology Co Ltd). 48 h of transfection later, we named AGS cells with steady *BLID* overexpression AGS/BLID-OE and those possessing empty vector as AGS-NC.

### RT-qPCR

The total RNAs were extracted with RNeasy Mini Kit. The Revert Aid First Strand cDNA Synthesis Kit together with miScript Reverse Transcription Kit were used for the reverse transcription of total RNAs into the complementary DNA (cDNA) of *RNU12*, hsa-miR-575, *PCNA, BCL-2, CCND1, E-cadherin, N-cadherin* and *Vimentin* in AGS cells. The miScript SYBR Green PCR Kit and LightCycler 480 SYBR Green I Master were used to assess the gene expressions using the resulted cDNAs as templates. The housekeeping gene β-actin was the internal control for *PCNA, BCL-2, CCND1, E-cadherin, N-cadherin* and *Vimentin*, and U6 small nuclear RNA was the internal reference for hsa-miR-575 expression. The 2^−ΔΔCt^ method served for calculating the relative gene expression^16,50^. The comparative 2^−ΔΔCt^ method helped to quantify the relative mRNA expression regarding *RNU12* in all GC samples. β-actin assisted in the normalization of *RNU12* expression^50,51^.

### MTT assay and cell cycle analysis

Cells were cultured overnight after transfection, then the collected cells were resuspended with the medium, and added into 96-well plates (5000 cells in 100 μl/well). The cell proliferation at 24 h, 48 h, 72 h, and 96 h later was measured by adding 20 μl/well of MTT solution (Promega, Madison, WI, USA) at each time point. After that, cells underwent four hours of incubation at 37 °C. then adding 150 μl of dimethyl sulfoxide into each well and shaking 10 min for completely dissolving the MTT. Model 680 reader was used for determining the absorbance exhibited by each well. The Flow Cytometer (Thermo Fisher Scientific, USA) assisted in analyzing the cell cycle, following the instructions of the manufacturer^52^.

### Transwell invasion assay (TIA)

The transwell chambers was used for assessing the cell migration. The cells were seeded in serum-free medium of the Matrigel-precoated upper-side of chambers (BD Biosciences, USA). The bottom chambers were added with the serum-free medium containing DMEM/F-12 without FBS as a chemoattractant. After 24 h, a cotton swab was employed for removing the residual non-invasive cells in the upper side of chambers. The cells moved to the low side of chambers were fixed with 100% methanol, then stained with 0.5% crystal violet. The transwelled cells were pictured under the inverted microscope.

### Colony formation assay

For colony-formation assay, 200 cells/well were seeded into six-well plates and cultured for 2 weeks, then the colonies were fixed in methanol, stained with 0.2% crystal violet and colonies with over 50 cells were photographed with Image Scanner (GE, USA) and analyzed by ImageJ (NIH, MD, USA).

### Wound healing assay (WHA)

The transfected cells were detached with Trypsin, suspended in DMEM/F12-10% FBS and seeded in 6 well plate in triplicates. 24 h later, a wound was inflicted in the midline of each well by a 10 μl sterile pipette tip. PBS was used for carefully washing the removed cells twice. 24 h later, the cells migrated in the midline were photographed under microscope and analyzed with Image J.

### Prediction of target genes of *RNU12* and hsa-miR-575

LncRNASNP2 database assisted in predicting the binding site for hsa-miR-575 in *RNU12*. The miRDB (http://mirdb.org/) and Target Scan (http://www.targetscan.org/vert_72/) were used for the analysis.

### Dual-luciferase reporter

113 pairs of GC and their adjacent normal gastric tissues came from tumor biobank. The wild-type (wt) *RNU12* and mutant (mut) *RNU12* were compared to predict hsa-miR-575-binding site which then chemically synthesized (Hanheng Biotechnology Co. Ltd) and inserted into pSI-check2 luciferase reporter plasmids for generating the pMIR-RNU12-wt (RNU12-wt) and pMIR- RNU12-mut (RNU12-mut) reporter plasmids. Similarly, BLID-wt and BLID-mut were also generated.

For reporter assay, cells in the 24-well plates cultured to 70% confluence, the reporter plasmids and hsa-miR-575 mimics or miR-NC were co-transfected with Lipofectamine 2000 reagent. 48 h later, Dual-Luciferase Reporter Assay System was used to detect the luciferase activity exhibited by the transfected cells. Normalization between relative luciferase activity and Renilla luciferase activity was achieved.

### RNA immunoprecipitation (RIP)

The RNA immunoprecipitation (RIP) was performed with EZ-Magna RIP Kit. Briefly, 1 × 10^7^AGS cells were lysed using RIP lysis buffer, mixed with magnetic beads coated with Anti-IgG or Anti-Ago2. After 6 h, these beads enriched RNAs of *RNU12* and miR-575 complex which were extracted and quantified with RT-qPCR.

### Western blot analysis

Cells harvested and lysed with RIPA buffer that contained 0.1% SDS and protease inhibitor cocktail. BCA protein assay was used for measuring the protein concentration of supernatants. 25 μg of proteins were run on SDS–PAGE (sodium dodecyl sulfate and 10–12% polyacrylamide gels) and transferred 1.5 h onto a nitrocellulose membrane (Millipore A) at 100 V. After blocking with 3% BSA in TBST (TBS-1% Tween 20) at room temperature, and the membranes incubated overnight at 4 °C with 1:1,000 primary antibodies, BLID(Abcam, Cambridge, MA), PCNA (Cell Signaling Technology,CA, USA), BCL-2 (Cell Signaling Technology,CA, USA), CCND1 (Cell Signaling Technology,CA, USA), E-cadherin (Cell Signaling Technology,CA, USA), N-cadherin (Cell Signaling Technology,CA, USA) and Vimentin (Cell Signaling Technology,CA, USA), respectively. After washed three times using TBS-N, the membranes were incubated with secondary horseradish peroxidase conjugated anti-rabbit IgG (1:2000; Cell Signaling Technology, CA, USA) for one hour at room temperature and stained with Immobilon ECL Ultra Western HRP Substrate. The Image Station 4000MM Pro imaging platform was used to reveal the bands. Protein levels relative to the load control β-actin were quantified by Image J.

### Transplantation of zebrafish with *RNU12* knockdown GC Cell AGS

Transgenic zebrafish TG (apo14-GFP) came from the Institute of Hydrobiology (Chinese Academy of Sciences). Adult fish were raised at 26.5 °C, with the light/dark ratio of 12 h:12 h. The IVF Development Zone of apo14-EGFP zebrafish yolk sac was injected with AGS/RNU12-SH cells and AGS/NC cells on the 2nd day following fertilization. Following the injection, embryos received 24 h of culturing at 33 °C, followed by being transferred to an incubator at 35 °C. A laser scanning confocal microscope (LSM 710) assisted in observing in detail how red fluorescence was distributed and expressed in zebra’s abdominal cavity. ImageJ 1.48v was used for analyzing red fluorescence expression in the abdominal cavity. An inverted SP5 STED confocal microscope was used for imaging and quantifying the results. Each group had no less than 40 zebrafishes, with three representative images being utilized. All the tests were parallelly carried out three times.

### Statistical analysis

SPSS 19.0 software assisted in data analysis. Pearson analysis was used for correlation. The two tailed *t* test served for examining group difference. Paired or nonparametric Kruskal–Wallis test served for evaluating how *RNU12* level was related to other characteristics. The Kaplan–Meier method calculated the survival curve. *P* value less than 0.05 (*P < 0.05, **P < 0.01, ***P < 0.001, and ****P < 0.0001) denoted statistical significance.

## Supplementary Information

Supplementary Figure 1.

Supplementary Figure 2.

Supplementary Figure 3.

Supplementary Figure 4.

Supplementary Information 5.

Supplementary Table 1.

Supplementary Table 2.

## Data Availability

All data generated or analyzed during this study are included in this published article.
