# Supplementary material for: RNU12 inhibits gastric cancer progression via sponging miR-575 and targeting BLID
Source: Sci Rep. 2023 May 9;13:7523. doi: 10.1038/s41598-023-34539-4 (PMC10169768; doi:10.1038/s41598-023-34539-4)
Supplement: Supplementary file 1 — Supplementary Figure 1. [file 41598_2023_34539_MOESM1_ESM.pdf]

Supplemental Figure 1

raw_images for Figure 2I

AGS

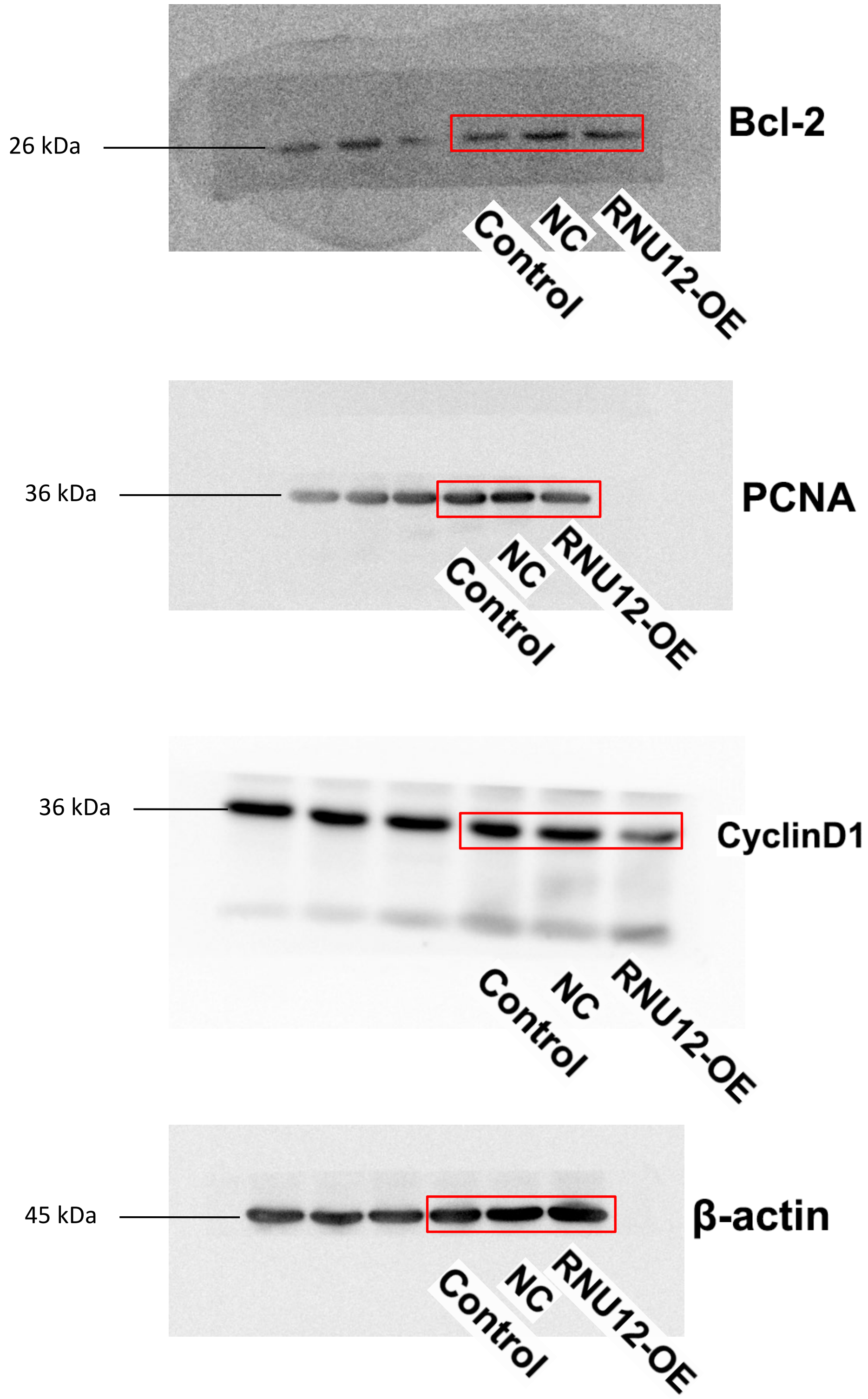

raw_images for Figure 2K

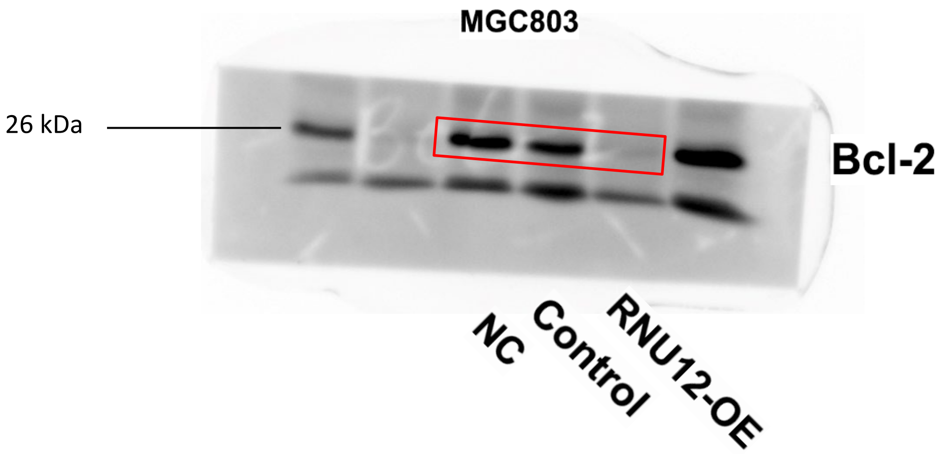

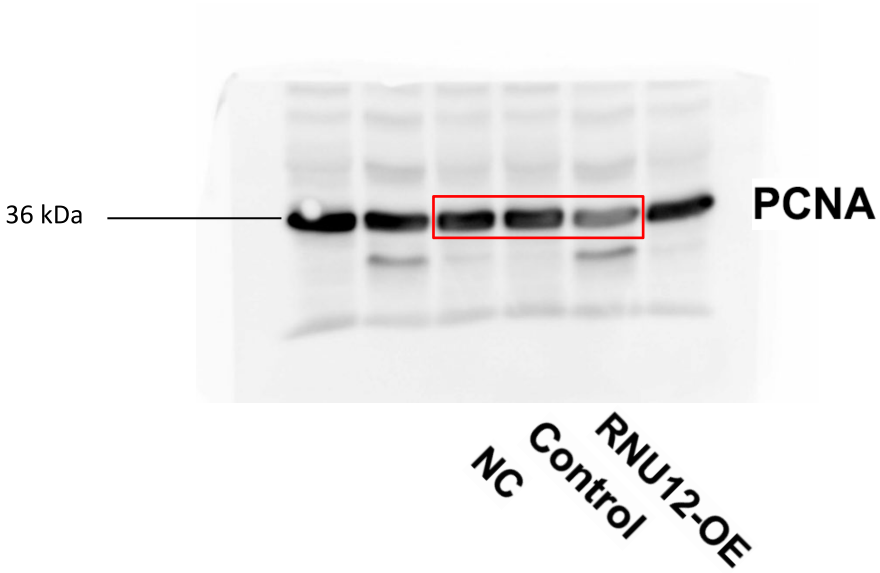

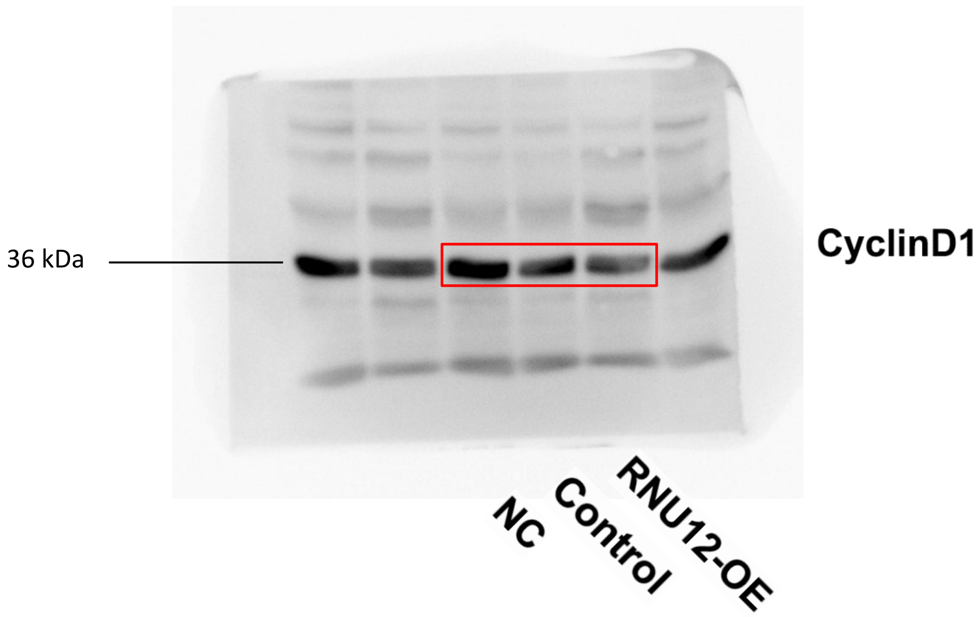

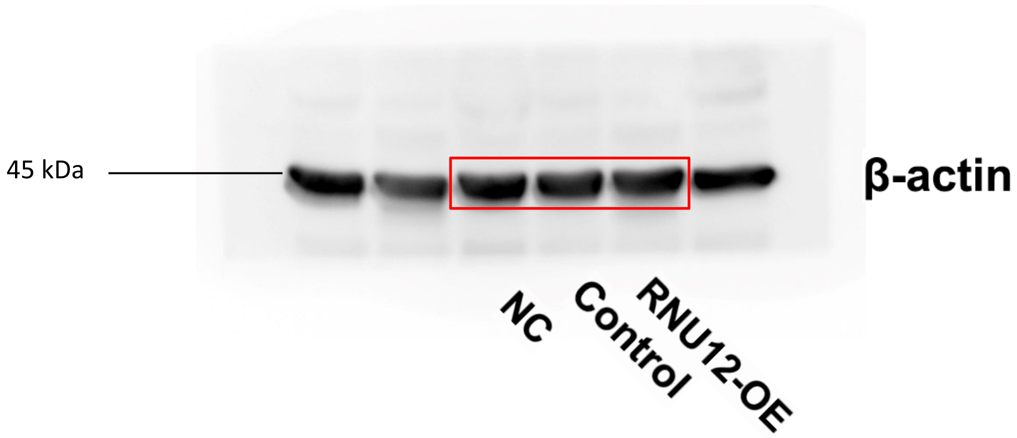

raw_images for Figure 2R

AGS

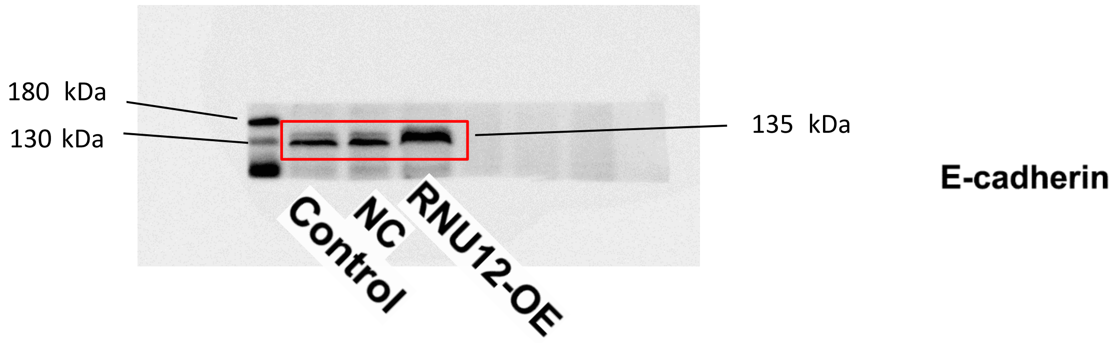

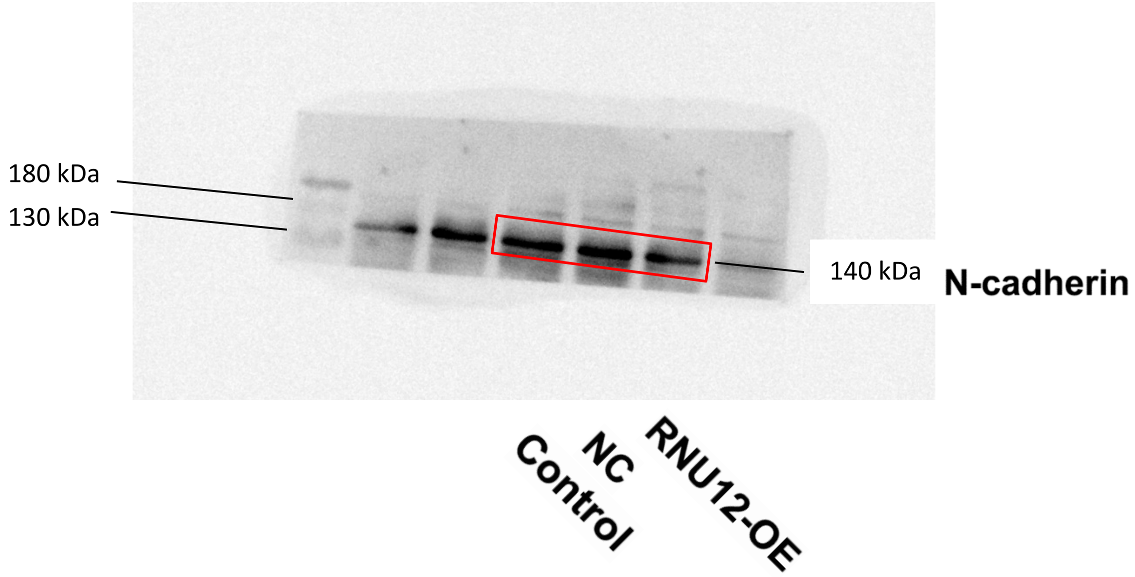

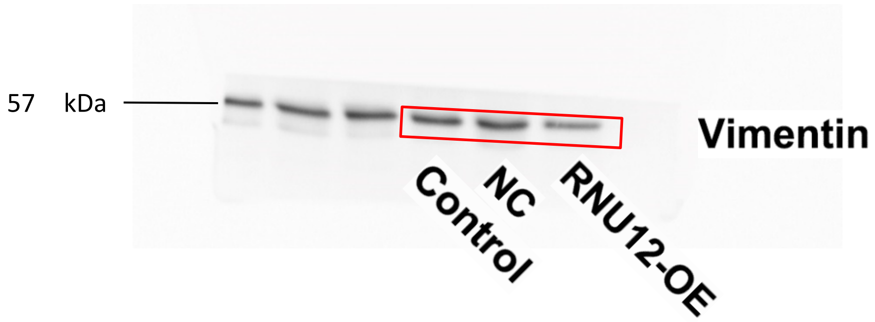

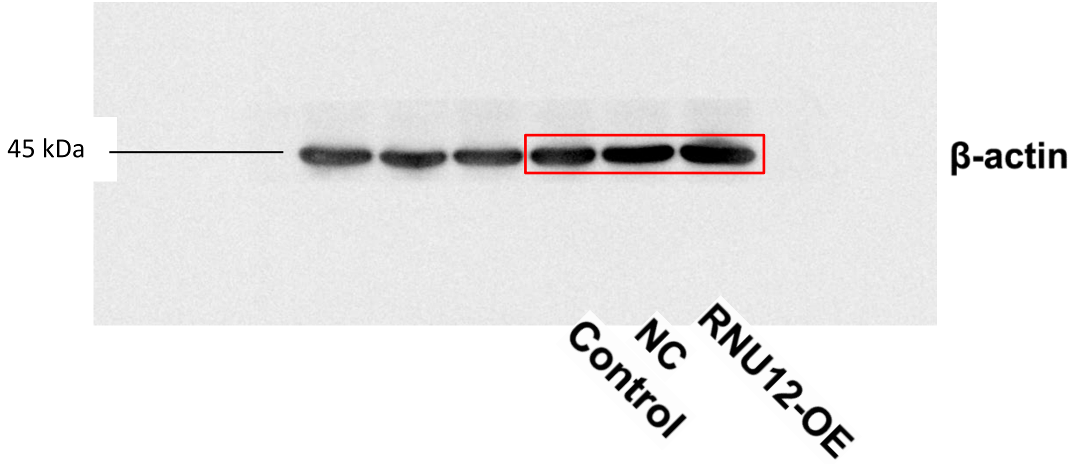

raw_images for Figure 2S

MGC803

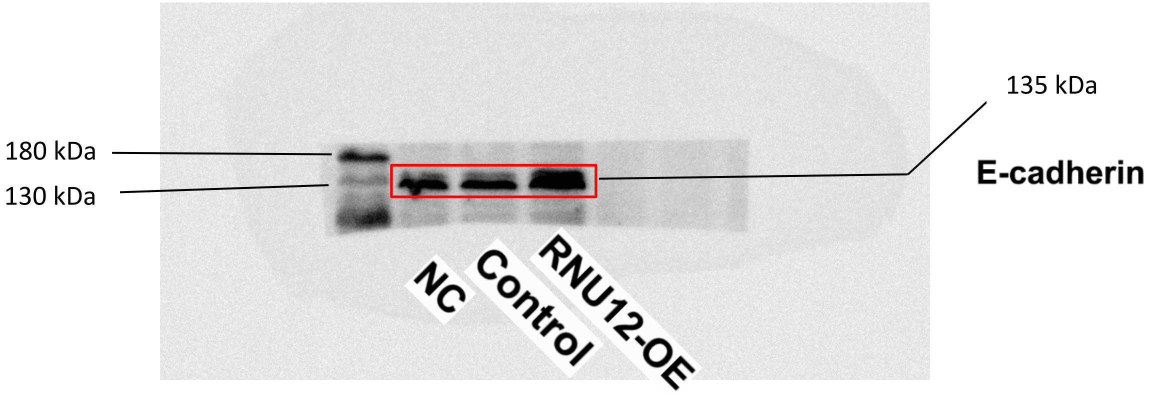

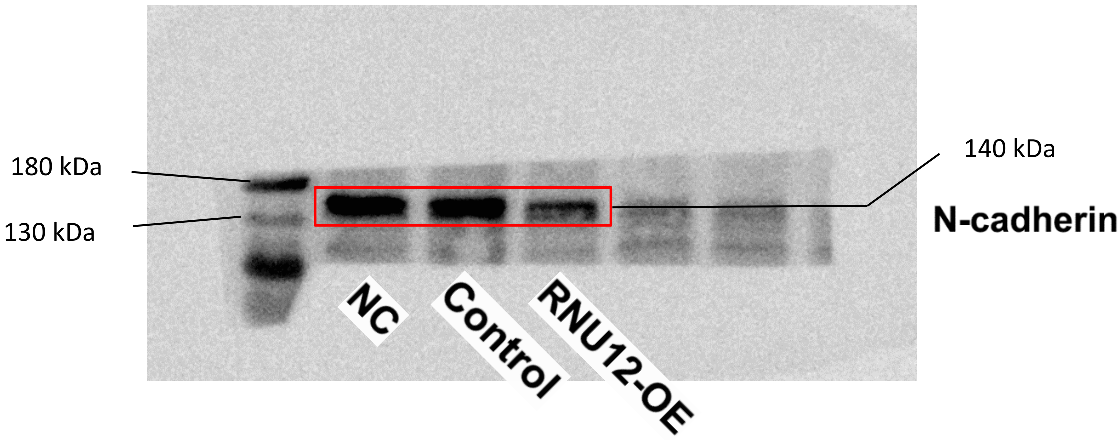
180 kDa
130 kDa
140 kDa
N-cadherin
NC
Control
RNU12-OE

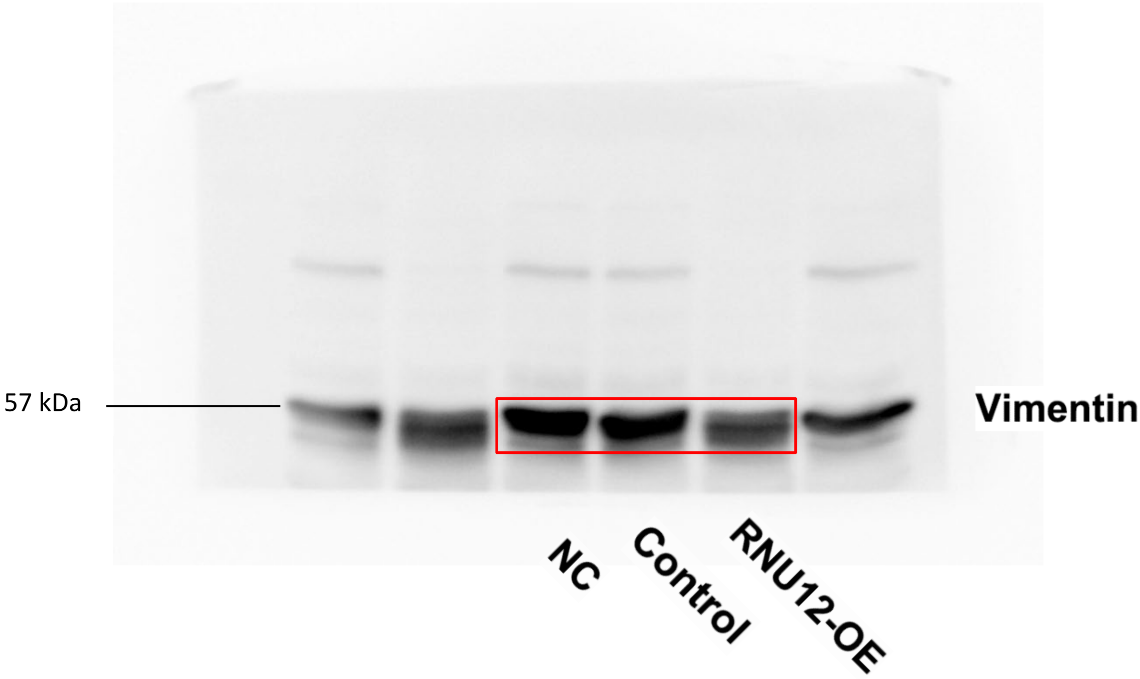
57 kDa
Vimentin
NC
Control
RNU12-OE

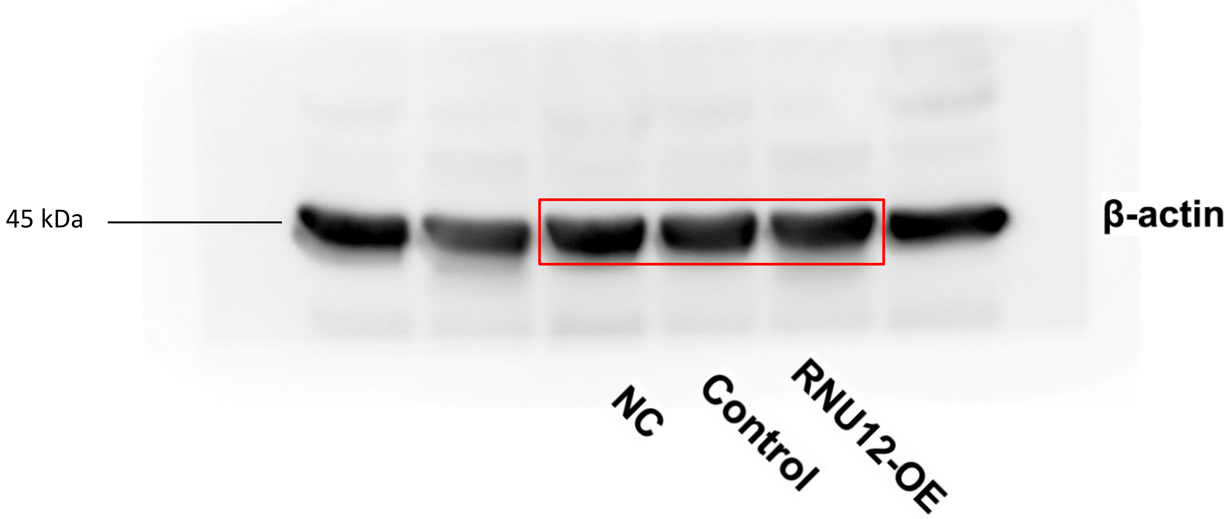
45 kDa
β-actin
NC
Control
RNU12-OE
