# Supplementary material for: RNU12 inhibits gastric cancer progression via sponging miR-575 and targeting BLID
Source: Sci Rep. 2023 May 9;13:7523. doi: 10.1038/s41598-023-34539-4 (PMC10169768; doi:10.1038/s41598-023-34539-4)
Supplement: Supplementary file 2 — Supplementary Figure 2. [file 41598_2023_34539_MOESM2_ESM.pdf]

Supplemental Figure 2

raw_images for Figure 3I

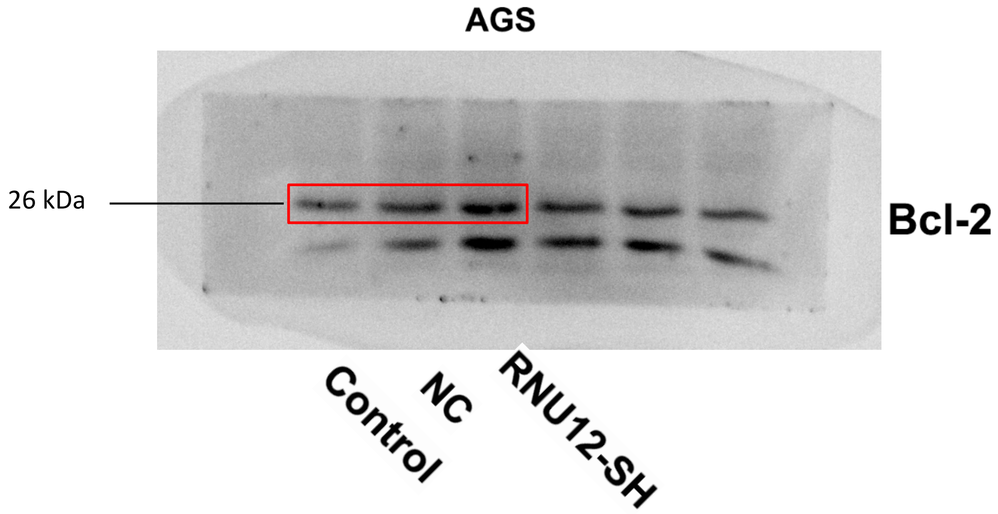

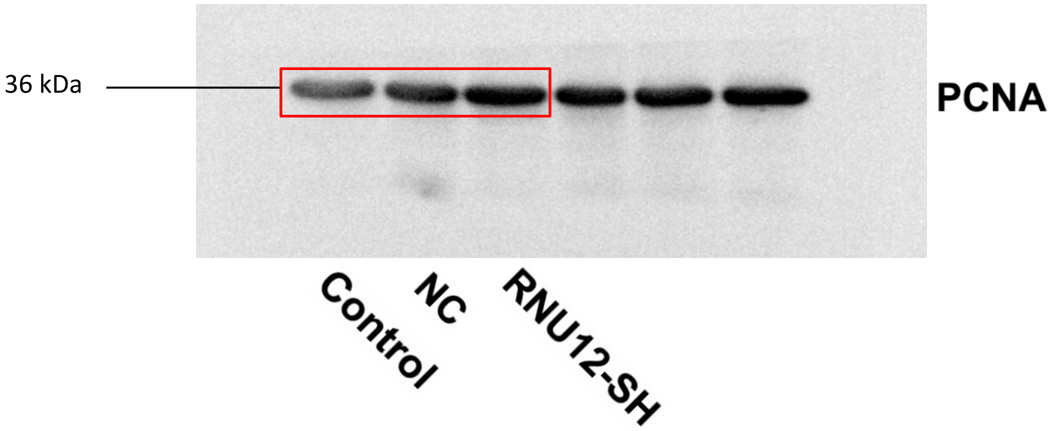

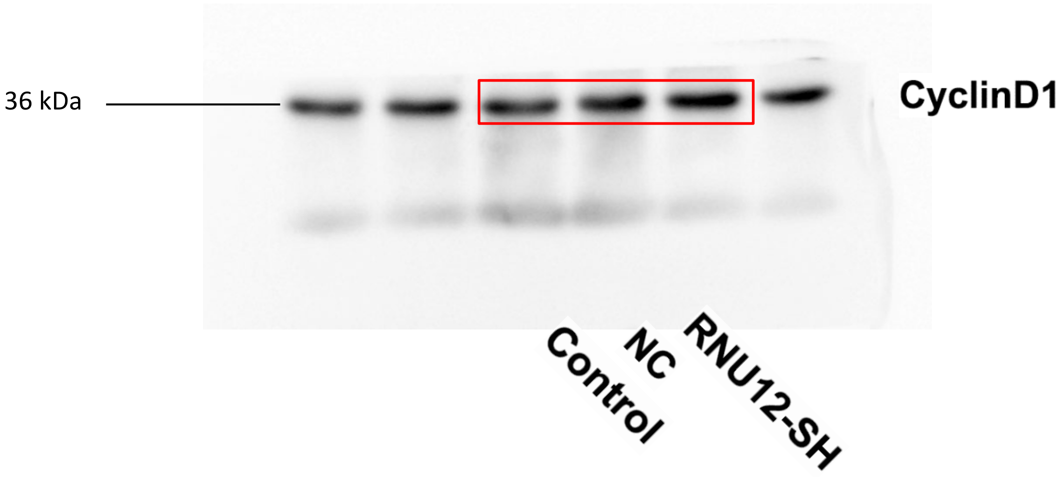

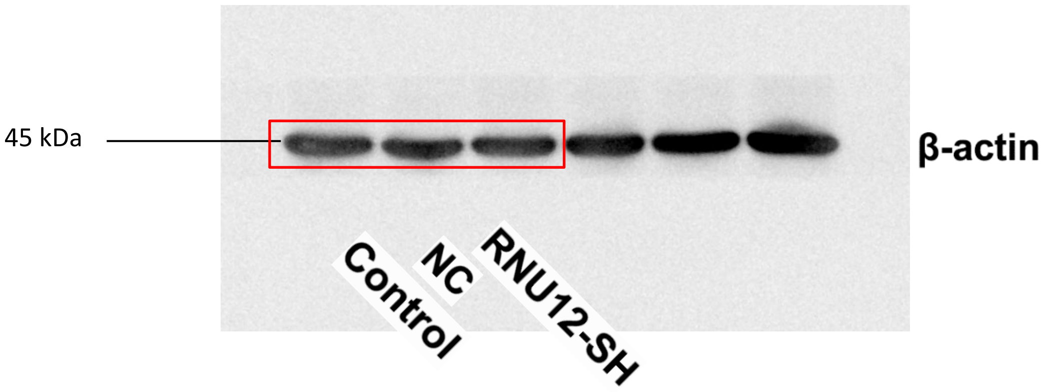

raw_images for Figure 3K

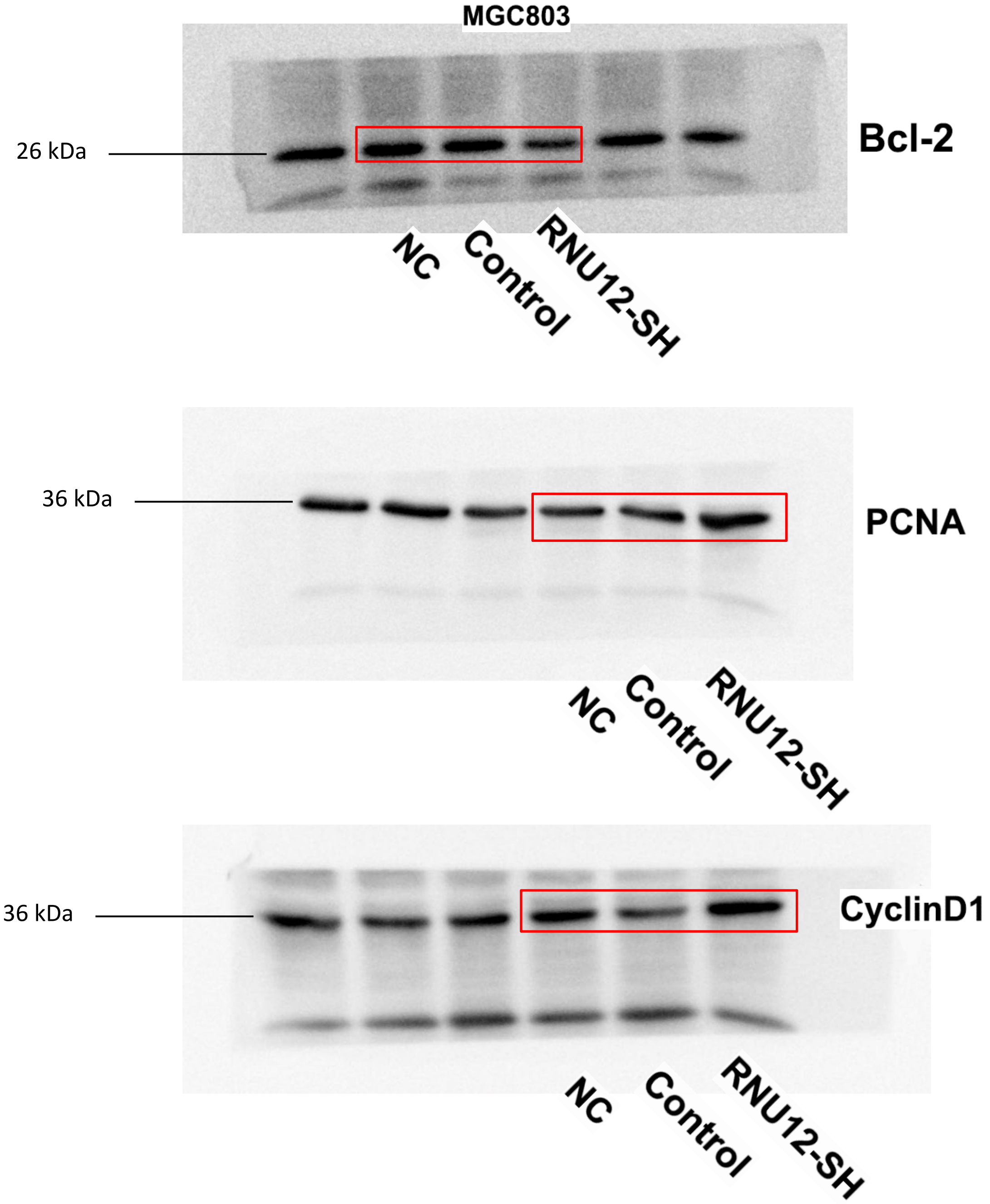

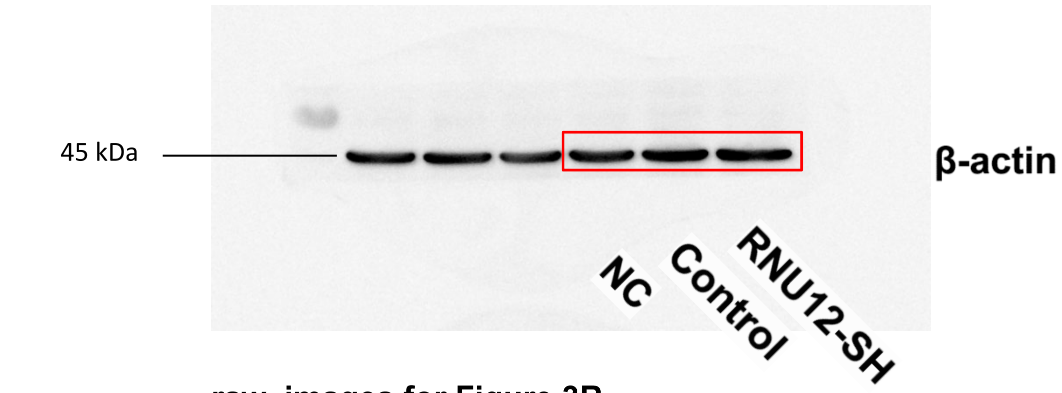

raw_images for Figure 3R

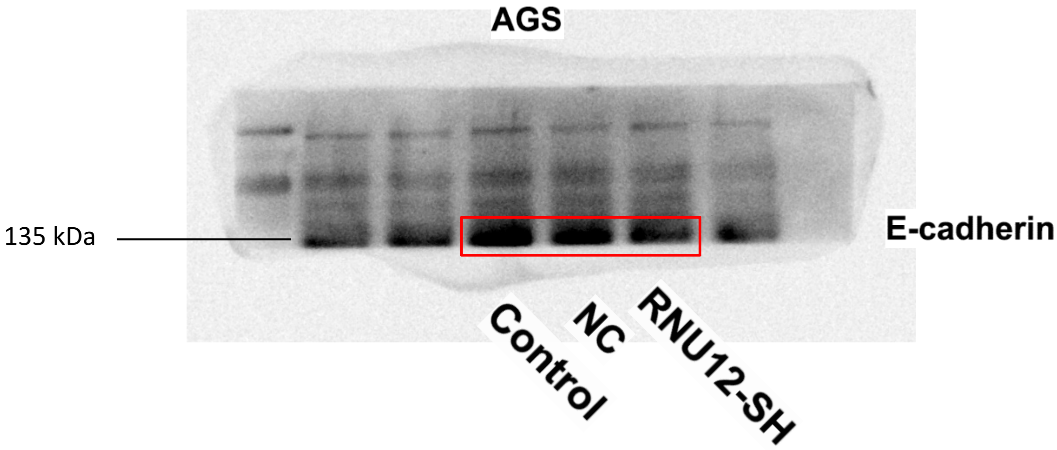

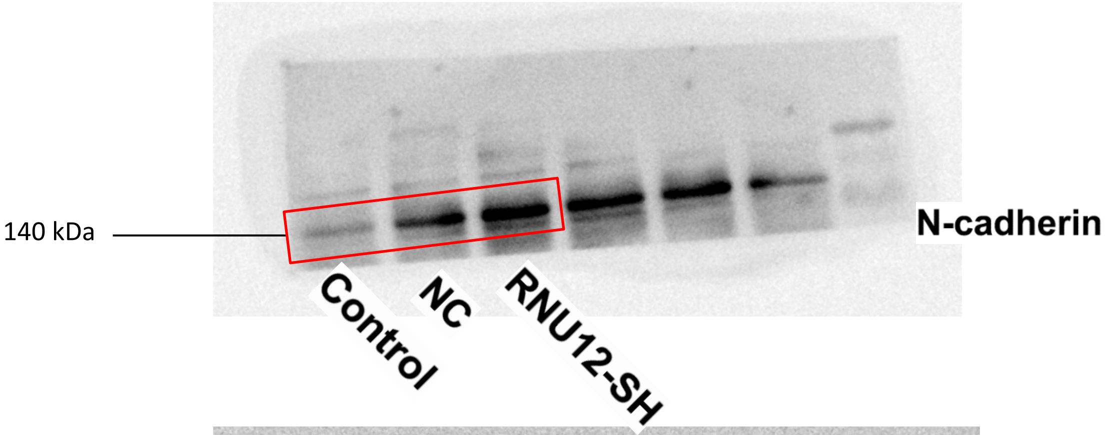

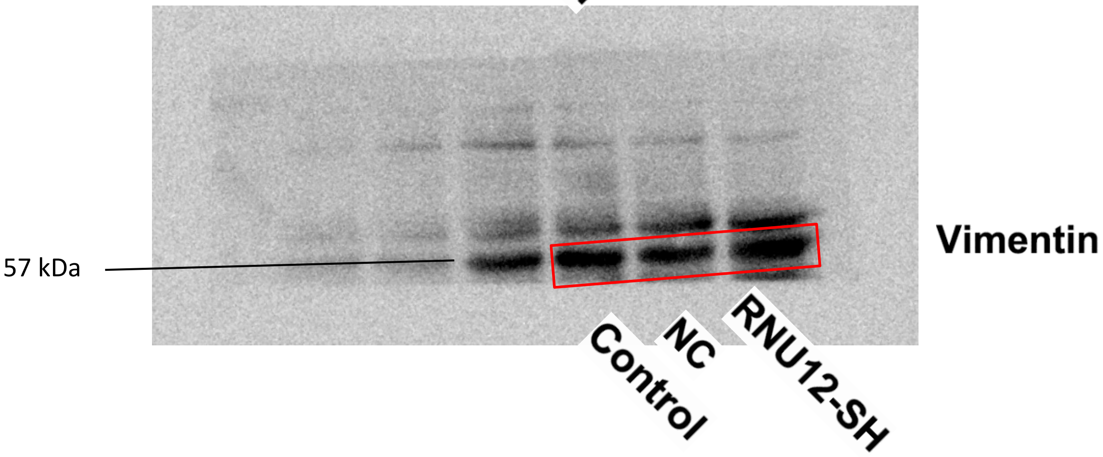

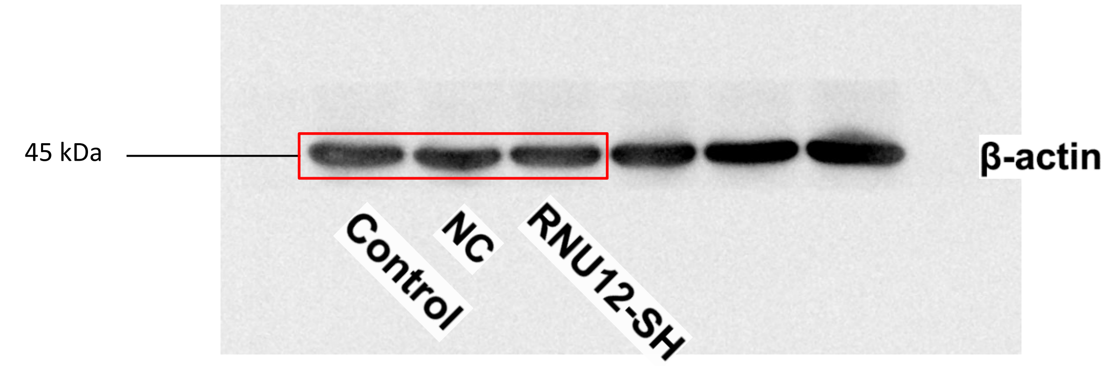

raw_images for Figure 3S

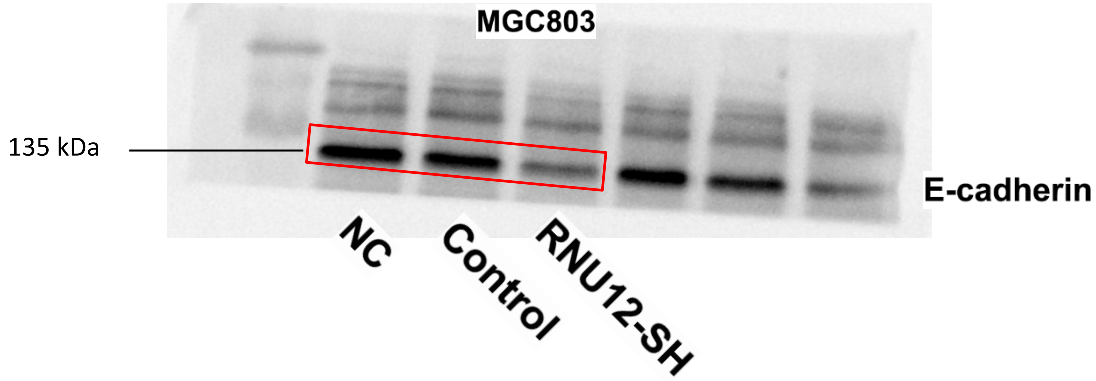

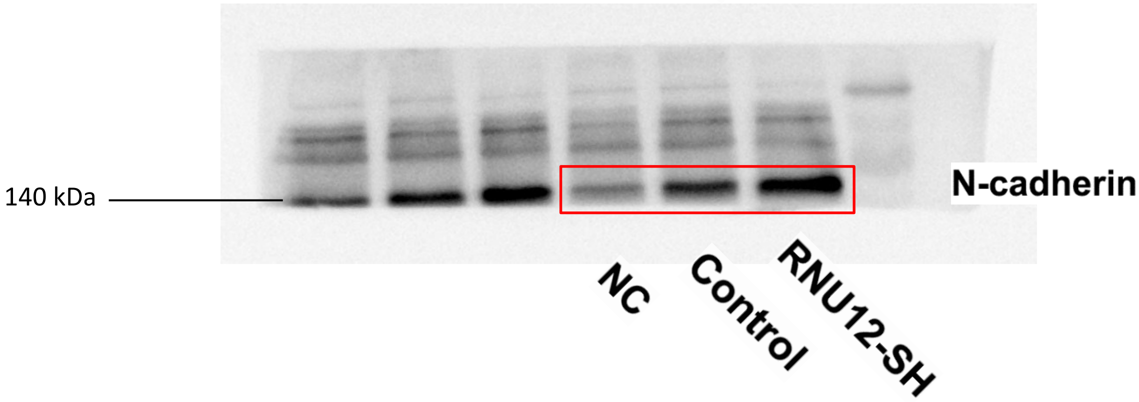

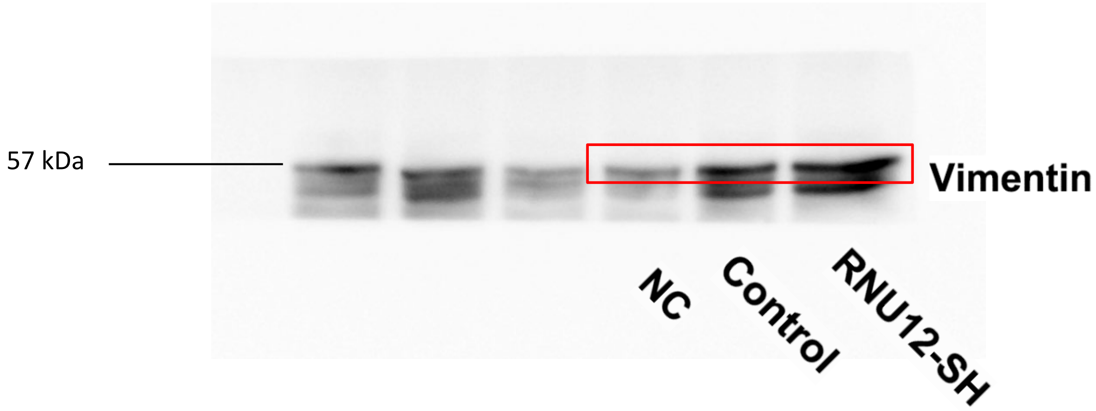

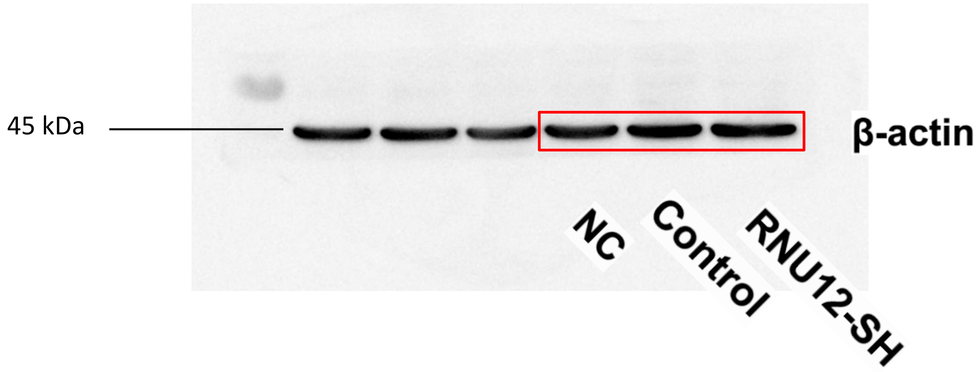
45 kDa
β-actin
NC
Control
RNU12-SH
