# Supplementary figures and images for: RNU12 inhibits gastric cancer progression via sponging miR-575 and targeting BLID
Source: Sci Rep. 2023 May 9;13:7523. doi: 10.1038/s41598-023-34539-4 (PMC10169768; doi:10.1038/s41598-023-34539-4)

### Supplemental Figure 3

raw\_images for Figure 4E

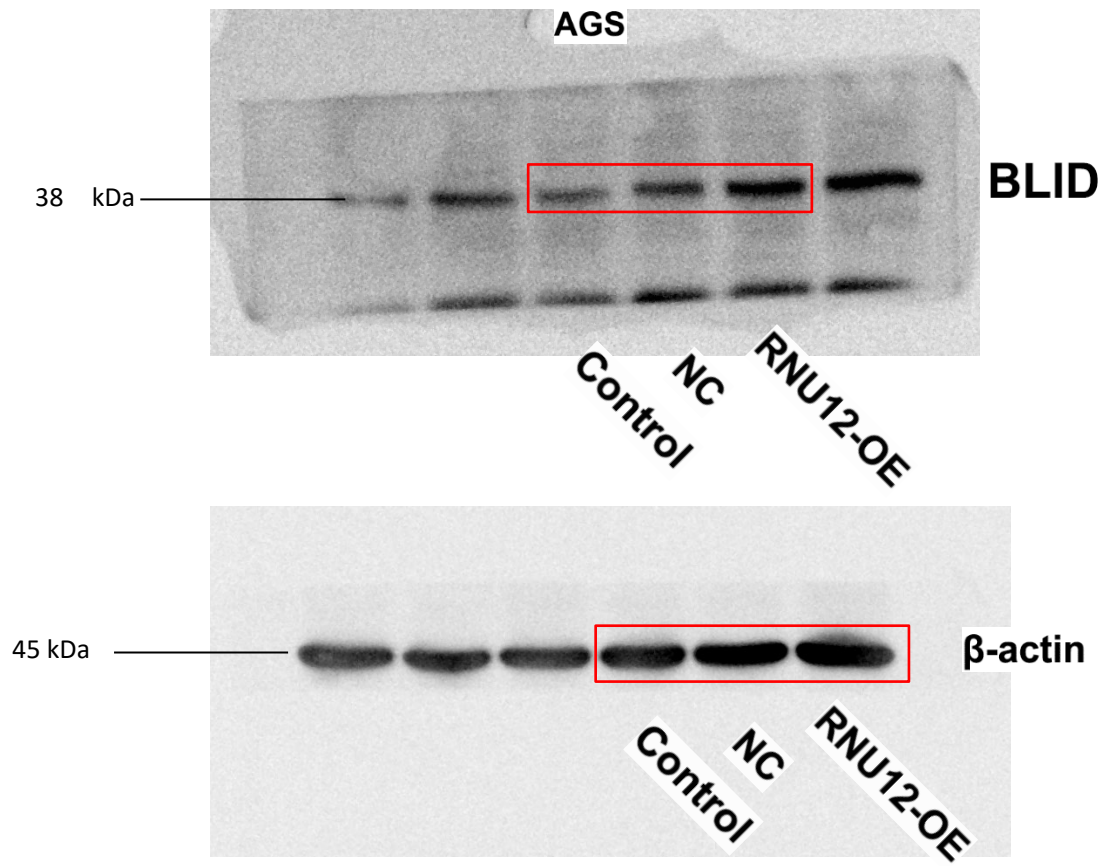

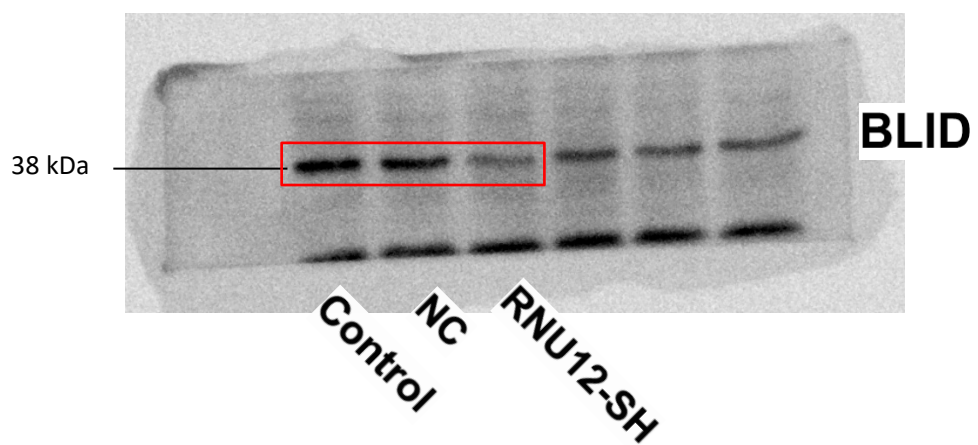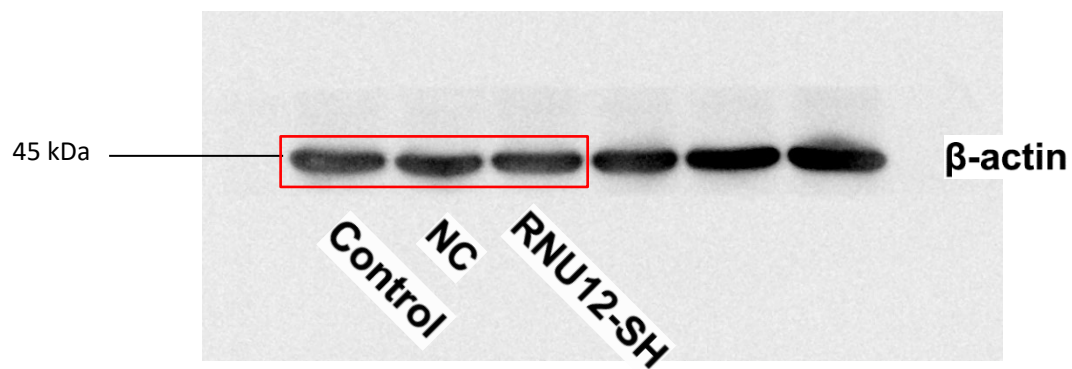

Supplement: Supplementary file 3 — Supplementary Figure 3. [file 41598_2023_34539_MOESM3_ESM.pdf]
