# Supplementary material for: RNU12 inhibits gastric cancer progression via sponging miR-575 and targeting BLID
Source: Sci Rep. 2023 May 9;13:7523. doi: 10.1038/s41598-023-34539-4 (PMC10169768; doi:10.1038/s41598-023-34539-4)
Supplement: Supplementary file 4 — Supplementary Figure 4. [file 41598_2023_34539_MOESM4_ESM.pdf]

Supplemental Figure 4

raw_images for Figure 5B

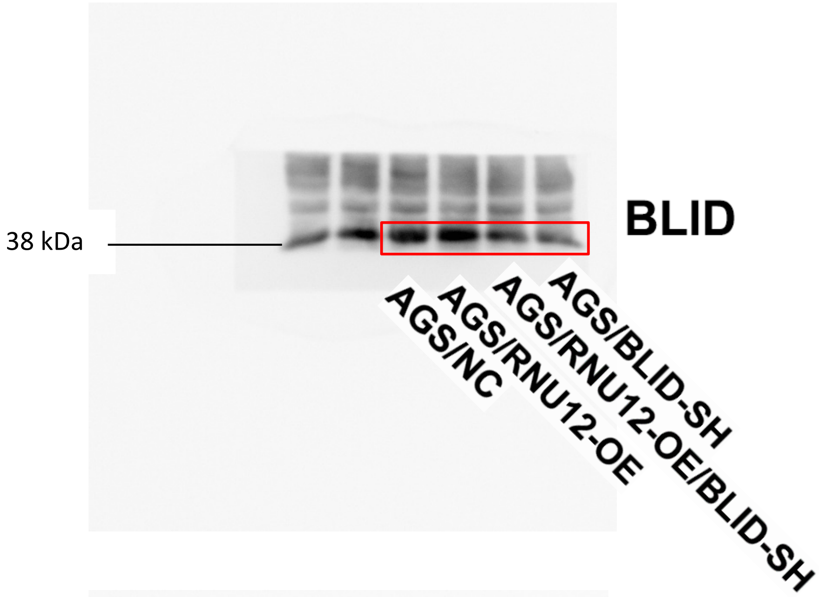

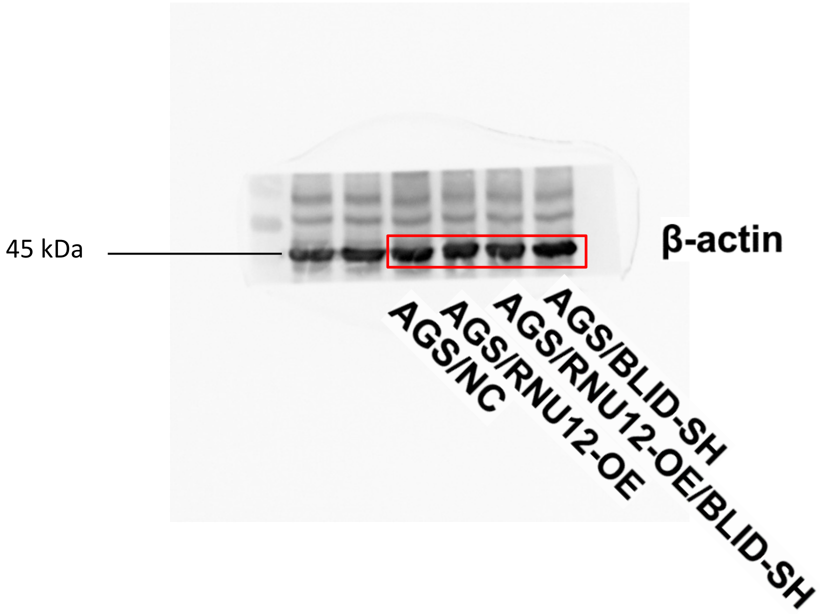

raw_images for Figure 5G

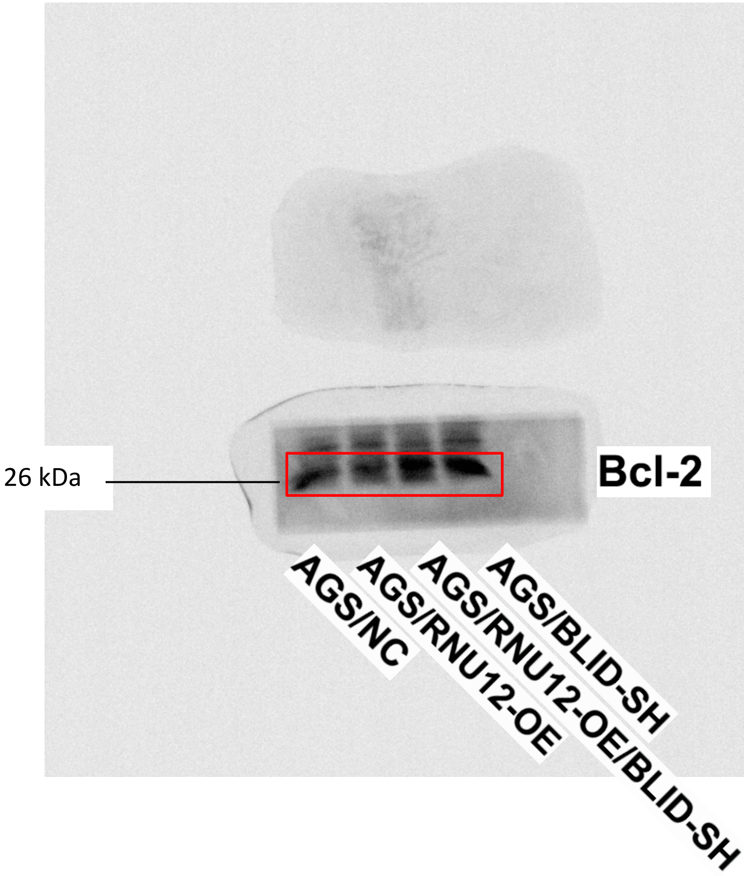

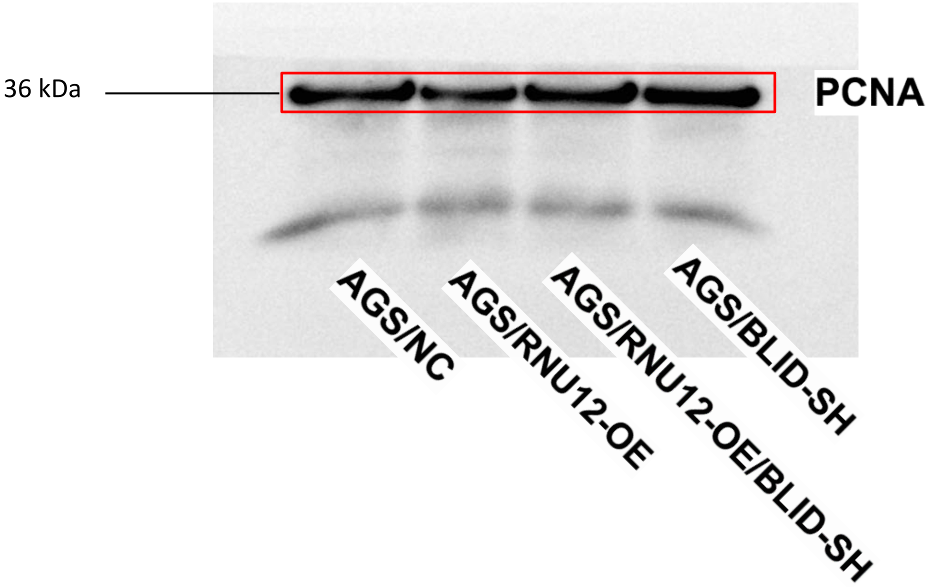

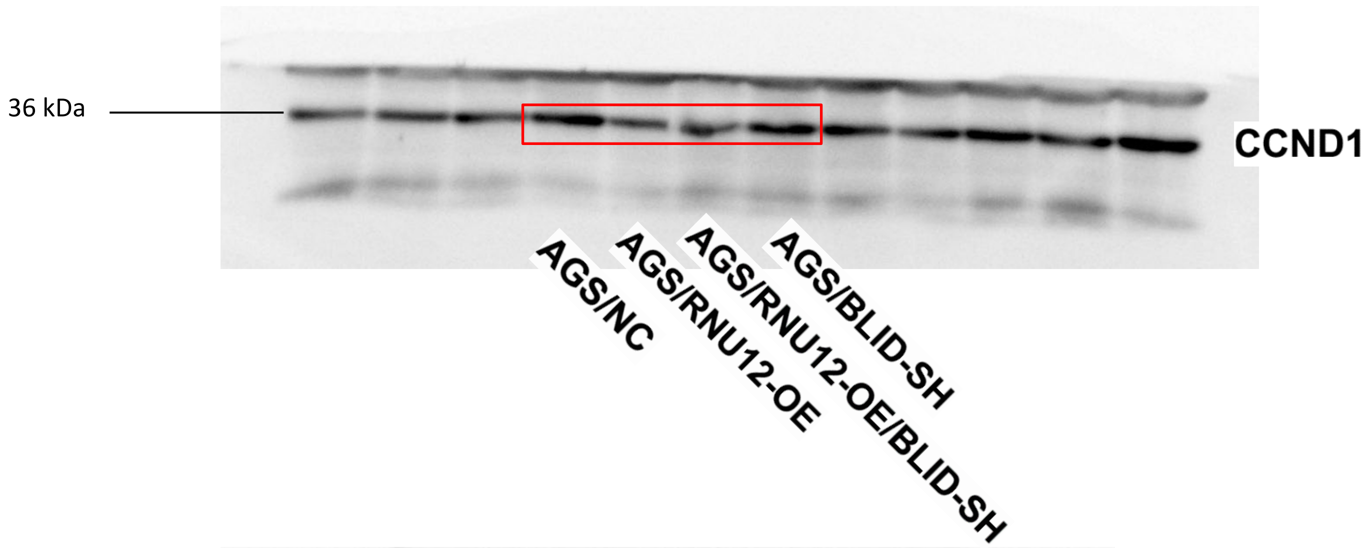

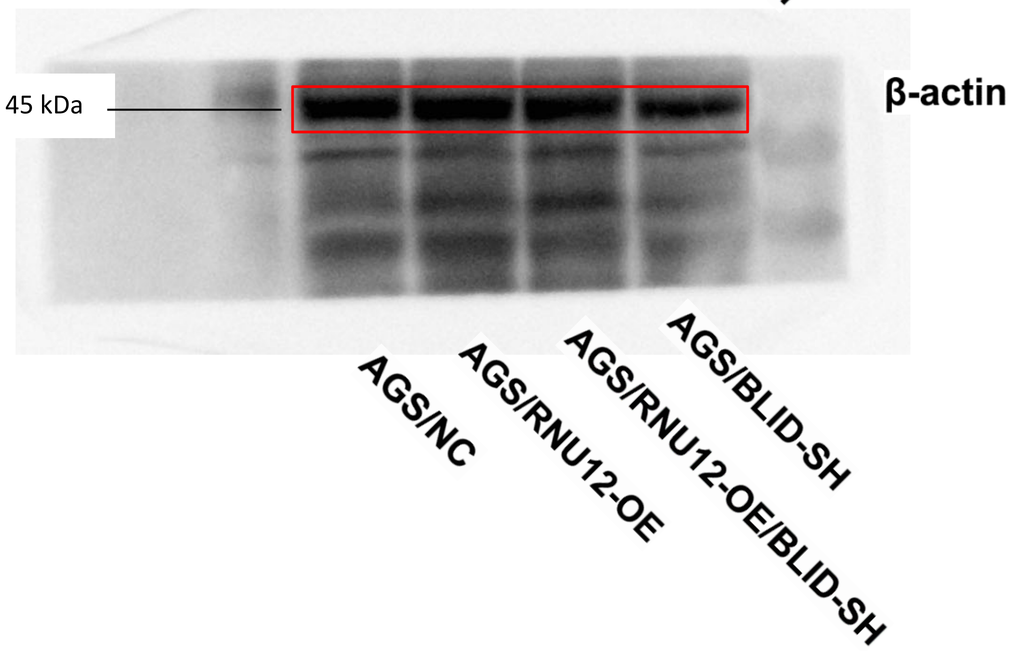

raw_images for Figure 5K

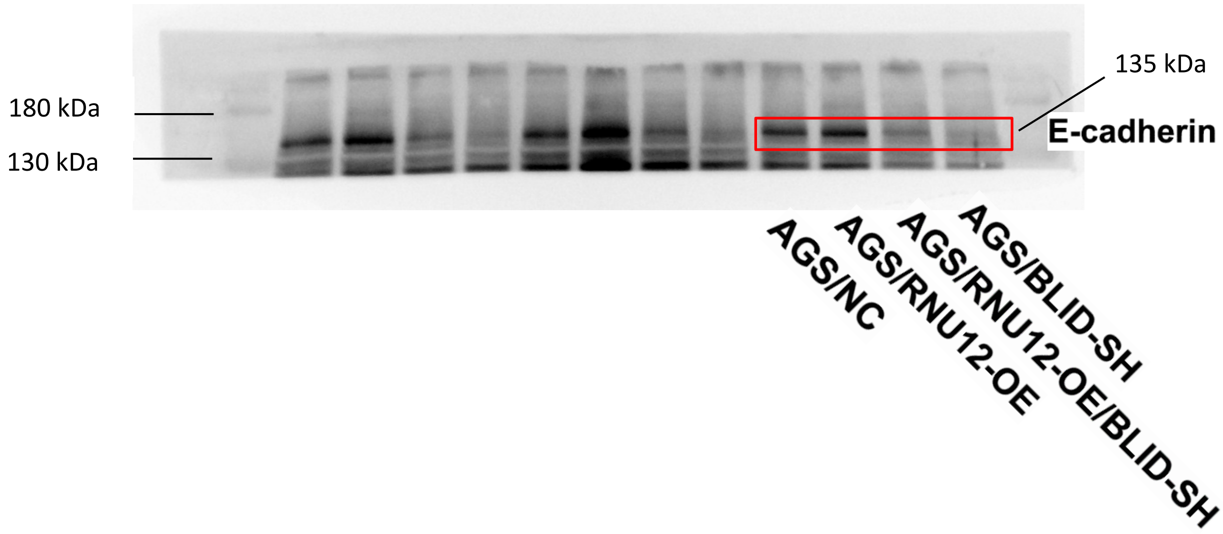

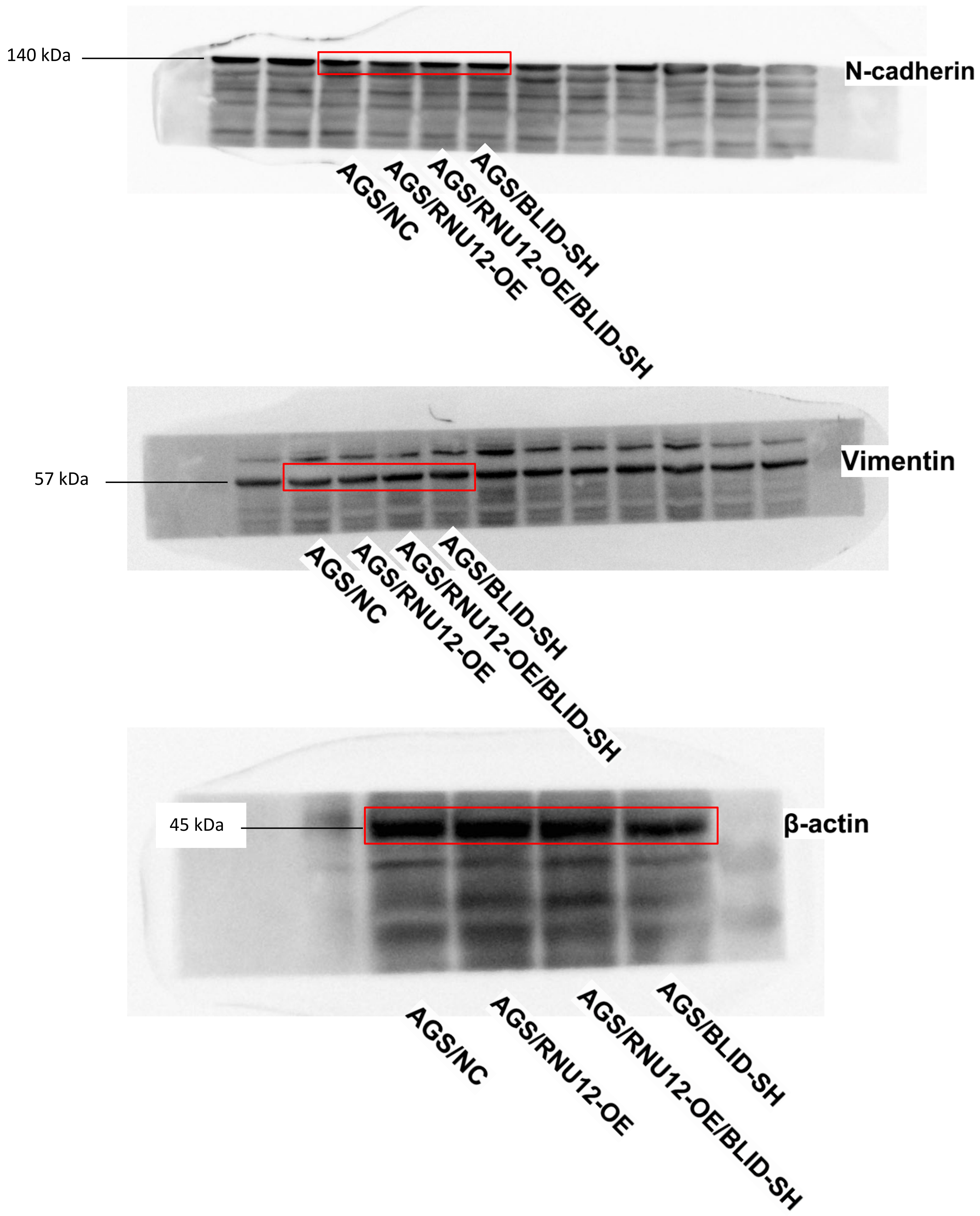

140 kDa
N-cadherin
AGS/NC
AGS/RNU12-OE
AGS/RNU12-OE/BLID-SH
AGS/BLID-SH
57 kDa
Vimentin
AGS/NC
AGS/RNU12-OE
AGS/RNU12-OE/BLID-SH
AGS/BLID-SH
45 kDa
β-actin
AGS/NC
AGS/RNU12-OE
AGS/RNU12-OE/BLID-SH
AGS/BLID-SH
