# Supplementary material for: RNU12 inhibits gastric cancer progression via sponging miR-575 and targeting BLID
Source: Sci Rep. 2023 May 9;13:7523. doi: 10.1038/s41598-023-34539-4 (PMC10169768; doi:10.1038/s41598-023-34539-4)
Supplement: Supplementary file 5 — Supplementary Information 5. [file 41598_2023_34539_MOESM5_ESM.pdf]

Recommendations for Loading

1. Thaw the ladder at room temperature for a few minutes to dissolve precipitated solids. Do not boil!
2. Mix gently, but thoroughly, to ensure the solution is homogeneous.
3. Load the following volumes of the ladder on an SDS-polyacrylamide gel:
   – 5 µL per well for mini gel,
   – 10 µL per well for large gel.
   Use the same volumes for Western blotting.
   The loading volumes listed above are recommended for gels with a thickness of 0.75-1.0 mm. The loading volume should be doubled for 1.5 mm thick gels.

Important Notes

- Prestained proteins can have different mobilities in various SDS-PAGE-buffer systems. However, they are suitable for approximate molecular weight determination when calibrated against unstained standards in the same system. See the table provided for migration patterns in different electrophoresis conditions.
- In low-percentage gels (< 10 %), the low-molecular weight proteins in the ladder may migrate with the dye front.
- PageRuler Prestained Protein Ladder can be used in Western blotting with all common membranes: PVDF, nylon and nitrocellulose.
- Longer transfer times or higher transfer voltages may be required for Western blotting of large (>100 kDa) proteins.

PageRuler Prestained Protein Ladder

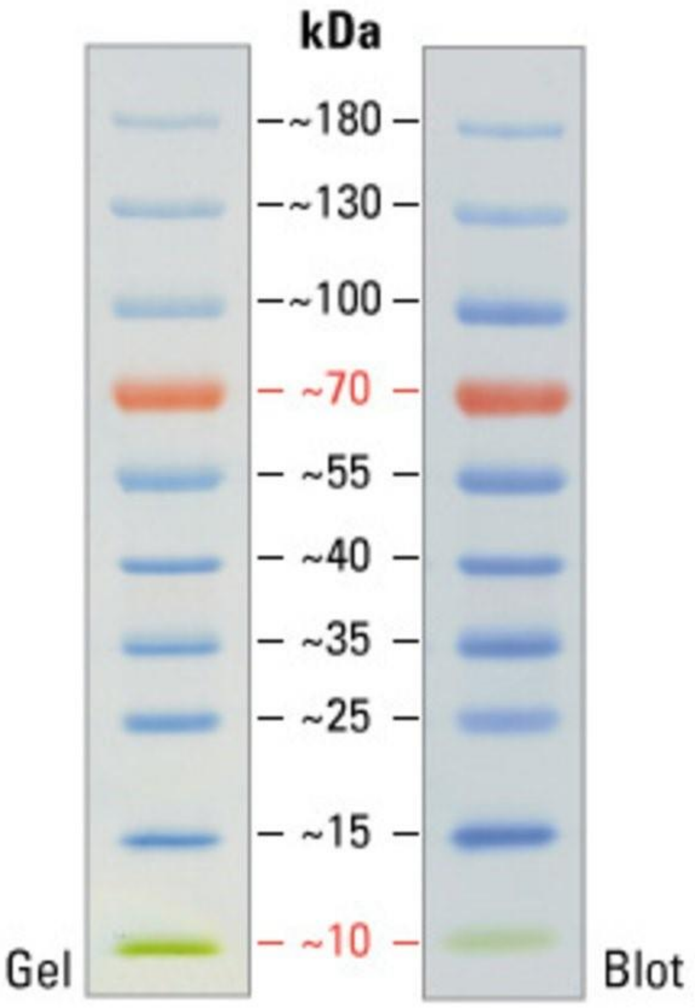

4-20% Tris-glycine SDS-PAGE
