# Supplementary material for: RNU12 inhibits gastric cancer progression via sponging miR-575 and targeting BLID
Source: Sci Rep. 2023 May 9;13:7523. doi: 10.1038/s41598-023-34539-4 (PMC10169768; doi:10.1038/s41598-023-34539-4)
Supplement: Supplementary file 6 — Supplementary Table 1. [file 41598_2023_34539_MOESM6_ESM.pdf]

Supplementary Table1. The list and sequence of long primers used for RT-qPCR analysis

Gene	Sequence（5'-3'）	Length(bases)
RNU12		
F-primer	AGACTGACTGTGGGGTGGTC	20
R-primer	GTGGGTCCCAACGTCAATAC	20
BLID		
F-primer	GCCTCTGGCAGTTCCATTTA	20
R-primer	CTGAAGCACATGTCCAGGAA	20
PCNA		
F-primer	GGCGTGAACCTCACCAGTAT	20
R-primer	TCTCGGCATATACGTGCAAA	20
CCND1		
F-primer	CTGCGAAGTGGAAACCATC	20
R-primer	CACATCTCCAGCATCCAGGT	20
BCL2		
F-primer	GCCTTCTTTGAGTTCGGTGG	20
R-primer	GAAATCAAACAGAGGCCGCA	20
